# Supplementary material for: Interplay of the forces governing steroid hormone micropollutant adsorption in vertically-aligned carbon nanotube membrane nanopores
Source: Nat Commun. 2024 Feb 6;15:1114. doi: 10.1038/s41467-024-44883-2 (PMC10847130; doi:10.1038/s41467-024-44883-2)
Supplement: Supplementary file 1 — Supplementary Information [file 41467_2024_44883_MOESM1_ESM.pdf]

Supplementary Information

Interplay of the forces governing steroid hormone micropollutant adsorption in vertically-aligned carbon nanotube membrane nanopores

Minh N. Nguyen [1], Melinda L. Jue [2], Steven F. Buchsbaum [2], Sei Jin Park [2], Florian Vollnhals [3], Silke Christiansen [3,4], Francesco Fornasiero [2], Andrea I. Schäfer [1] *

[1] *Institute for Advanced Membrane Technology (IAMT), Karlsruhe Institute of Technology (KIT), Hermann-von-Helmholtz-Platz 1, 76344 Eggenstein-Leopoldshafen, Germany*

[2] *Physical and Life Sciences Directorate, Lawrence Livermore National Laboratory (LLNL), 7000 East Avenue, Livermore, California 94550, United States*

[3] *Institute for Nanotechnology and Correlative Microscopy - INAM, Äußere Nürnberger Str. 62, 91301 Forchheim, Germany*

[4] *Fraunhofer Institute for Ceramic Technologies and Systems - IKTS, Äußere Nürnberger Str. 62, 91301 Forchheim, Germany*

*Corresponding author: andrea.iris.schaefer@kit.edu (A. I. Schäfer), +49 (0)721 6082 6906

Supplementary Discussion 1. Assumptions for forces acting in the nanochannel

The following discussion summarises the assumptions relevant for calculating the forces acting on the steroid hormone (SH) molecule, such as 17β-estradiol (E2) inside the carbon nanotube (CNT) channel.

1.1. The continuum theory in the 1.7−3.3 nm nanopores

- The continuum hydrodynamic framework does not break down for CNT channel size >1 nm, [1] so equation (3) in the main text used to calculate the (viscous) hydrodynamic drag force is applicable.

- The ethanol present at 15–30 mg L^{-1} concentrations in the feed solution is considered a part of the continuum.
- The viscosity of water does not deviate from the bulk in ~2 nm CNT channels. [2, 3]

1.2. Hydrodynamic drag force

- Coulombic drag caused by water flow in the nanochannel [4-6] is excluded because the solute (SH) is uncharged.
- The hydrodynamic drag force is the result of the viscous friction force acted by the water flow on the SH.
- SHs (*e.g.* E2) are treated as spherical molecules, with the hydrodynamic diameter of 0.8 nm. [7] This assumption allows the same projection area of E2 in all directions. In reality, SHs (*e.g.* E2) are not spherical but elongated, with the estimated width, height and length of 0.5, 0.6 and 1.1 nm (measured from the molecular structures with Chem3D software [8] (PerkinElmer Informatics, USA).
- Plug-like flow behaviour in the VaCNT membrane pore is satisfactory given the high slip length compared with the channel radius. As a result, the water flow velocity at the fluid–wall interface is equal to that in the pore centre. [9, 10]
- With the no-slip condition (*e.g.* in UF/NF membrane pores), the water flow velocity at the wall is zero and the flow velocity in the pore centre is highest, hence the drag force reduces drastically at the wall.
- When the SH is bound to the CNT wall, shear forces arise because the lateral water flow that drives the SH movement is opposed by the static CNT wall that resists SH movement.
- The wall correction factor that can yield an increased drag force is not considered assuming the slip condition. The flow is considered ‘unbound’, and the wall has no impact on the flow. [11]
- From the literature, when the no-slip condition was applied with more macroscopic systems (where the spheres and pores that are micrometres in diameter), the wall effect relevant to a sphere-to-pore diameter ratio similar to this study (0.4–0.5) can yield an increase in drag force of 2–6 times. [11, 12]

1.3. Adhesive (van der Waals) force

- The adhesive force F_A at the CNT surface is the primary non-directional van der Waals interaction. [13]

- The Hamaker constant (see equation (4) in the main text) is independent of the geometries of the SH and CNT wall. In reality, some specific groups (namely the π-rings) of the two species may form stronger interactions. [14]
- When the molecule is at the liquid−wall interface, F_A is balanced by the repulsive force caused by the repulsion F_R between the electrons of the SH and the electron of the CNT. This force balance results in the 0.32 nm gap on the CNT surface, as below this distance repulsion occurs ($F_\mathrm{R} > F_\mathrm{A}$). [15]
- The Hamaker constant specific for the interaction between an SH molecule and the CNT pore wall is corrected for the water medium [16] according to equation (S1).

$$H = \left(\sqrt{H_\mathrm{CNT}} - \sqrt{H_\mathrm{water}}\right)\left(\sqrt{H_\mathrm{SH}} - \sqrt{H_\mathrm{water}}\right) \quad \text{(S1)}$$

  where H_CNT, H_water, and H_SH are the Hamaker constants for the pair-wise interaction between two identical CNT, water, and SH molecules, respectively.
- The Hamaker constant for water−water interaction H_water [16] is reported to be $3.7 \cdot 10^{-20}$ J.
- $H_\mathrm{CNT} = 1.8 \cdot 10^{-19}$ J for SWCNT with an external diameter of 2 nm, [17] although this value is given for the external surface instead of the internal surface. It should be noted that the curvature inside the CNT pore was neglected, which results in an overestimation of van der Waals interaction.
- H_SH has not been reported, although for non-conducting liquids, [18] the Hamaker constant takes the values of $(5–10) \cdot 10^{-20}$ J.
- Based on the above values of H_CNT, H_water, and H_SH, H is then approximated to be $3.5 \cdot 10^{-20}$ J. This value is then applied in the adhesive force calculation with equation (4), main text.
- In reality, the CNT−hormone affinity is affected by hydrophobicity, polarity of the CNT wall and the SH molecules, [19] and the property of the medium that separates the SH and CNT wall. Hence, a uniform H value does not provide the correct determination of adhesive force.
- With the assumption of a uniform H, at a fix distance between the CNT and SH (for example, 0.32 nm), F_A is then dictated by the SH diameter, as such molecules with larger diameters result in stronger F_A.

The balance between the repulsive (F_R) and adhesive forces (F_A) can be visualised through the Lennard−Jones-type potential in a much simpler system consisting of two atoms (Supplementary Figure 1). [20] When these two atoms (*e.g.* one of the hormone molecule and one of the CNT wall) are very close to each other, the electrons of these atoms repel each other and generate a positive potential

energy, although this is countered by the negative potential energy due to attraction/adsorption ($F_A < F_R$). The repulsion energy vanishes at a shorter distance than the adhesion energy. The total potential energy reaches a minimum (where $F_R = F_A$) at a separation distance of ~0.3 nm. [15] When the hormone atom is further from the wall atom, $F_A > F_R$ and the hormone atom is driven towards the wall. The power scales of the attractive and repulsive potentials will deviate if the system now involves multiple atoms in the hormone molecule and multiple atoms in the CNT wall.

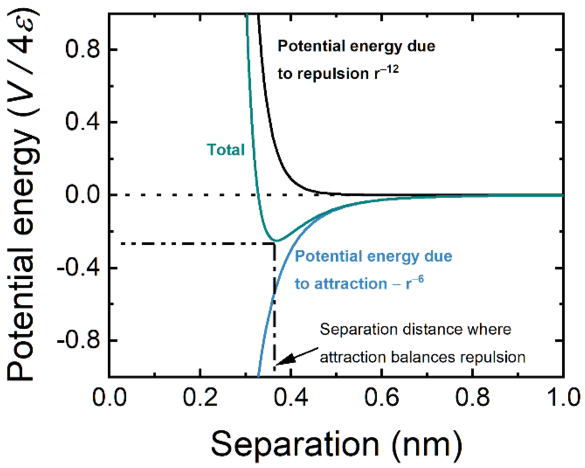

Supplementary Figure 1. The Lennard–Jones potential energy of the interaction between two atoms as a function of the separation distance. Adapted from Atkins, de Paula, and Keeler. [20]

1.4. Friction force

- The *viscous friction* applied to characterise fluid dynamics in the CNT pores [6, 21, 22] is formed when the surrounding solvent (water) molecules or the pore wall inflict resistance on the moving solvent or solute molecule.
- This viscous friction is distinguished from the friction that must be exceeded for an adsorbed molecule to start moving. [23] This second type of friction is referred to in the study as this study investigates the transition of the SH molecule from the static (adsorbed) state to the dynamic state.
- The surface of CNTs is considered perfectly smooth and defect-free, which allows an ultra-low friction of molecules on this surface. [24]

1.5. Dependence of the forces on material properties

From the above explanations and assumptions of the forces, the dependence of these forces on material properties (both the adsorbent surface and neutral adsorbate molecule) in the water medium is summarised in Supplementary Table 1.

Supplementary Table 1. Material properties that affect the force magnitude. Note: The adhesive and repulsive forces depend on the solute−surface distance.

**Force**	**Material properties that affect the force**	
	**Neutral solute (SH)**	**Surface**
Adhesive (van der Waals) F_A	Geometry<br>Orientation / polarity<br>Hydrophobicity<br>Dimension (or hydrodynamic diameter)	Roughness (nanoscale)<br>Hydrophobicity<br>Curvature (e.g. CNT diameter)
Repulsive F_R	Geometry<br>Orientation / polarity<br>Hydrophobicity<br>Dimension (or hydrodynamic diameter)	Roughness (nanoscale)<br>Hydrophobicity<br>Curvature (e.g. CNT diameter)
Hydrodynamic drag F_H	Geometry<br>Orientation in medium (water) / polarity<br>Dimension (or hydrodynamic diameter)	Hydrophobicity<br>Roughness (nanoscale)<br>Tortuosity (pore-size scale)
Friction F_F	Geometry<br>Orientation / polarity<br>Hydrophobicity<br>Dimension (or hydrodynamic diameter)	Roughness (nanoscale)<br>Hydrophobicity<br>Curvature (*e.g.* CNT diameter)

Supplementary Discussion 2. Enhanced flow in VaCNT membranes

2.1. Determination of water flow velocity in VaCNT, UF and NF membranes

A summary of membrane properties and experimental conditions in the comparison of SH (E2) adsorption is provided in Supplementary Table 2. The UF membrane data at 1 bar and NF membrane data at 5 bar were taken from Nguyen *et al.* [25] and Imbrogno and Schäfer, [26] respectively.

Supplementary Table 2. Brief summary of the properties of VaCNT, UF and NF membranes, and the experimental conditions (flux / pressure) for the comparison of SH adsorption.

**Parameter**	**Units**	**Membrane**		
		**VaCNT**	**UF**	**NF**
Origin (type, brand, company)		Fabricated [27]	UF 10 kDa, PLHGC, Millipore, USA	NF270, Dupont, USA
Pore diameter	nm	1.7 ± 0.7	5.4 [a]	0.8 ± 0.1 [b, 28]
Separation layer thickness	µm	43−57	0.5−1 [29]	0.02−0.04 [30, 31]
Total membrane thickness	µm	43−57	230 [32]	130 [30, 31]
Membrane porosity	%	3.4 ± 2.0 [c]	18 ± 4 [c]	3 ± 1 [7, 33]
Pure water permeability	$L\ m^{-2}\ h^{-1}\ bar^{-1}$	8−64	87 ± 8 [25, 34]	14 ± 2 [35]
Water flux in the experiment	$L\ m^{-2}\ h^{-1}$	57 ± 3	88 ± 5<br>(at 1 bar)	80 ± 10<br>(at 5 bar)

Avg. water flow velocity in a membrane pore [d]	10^{-4} m s^{-1}	4.7 ± 1.5	1.3 ± 0.3	7.4 ± 2.2
Average hydraulic residence time in the separation layer [e]	S	0.11 ± 0.07	0.007 ± 0.003	(5 ± 3) · 10^{-5}

[a] Calculated from the membrane molecular weight cut-off (10 kDa) following Worch [36]
[b] Calculated based on the steric hindrance pore model described by Deen [37]
[c] Estimated from the Hagen−Poiseuille equation (equation (1) in the main text) with a tortuosity correction factor equal to 1 [38]
[d] Calculated from the flux and porosity *via* equation (S14)
[e] Calculated from the flow velocity and separation layer thickness *via* equation (S15)

The experimental fluxes in the comparison between the VaCNT, UF and NF membranes vary within a relatively small range, between 60 and 90 L m^{-2} h^{-1}. With this flux range, the flow velocity in the respective membrane pores varies within an order of magnitude, from 1 · 10^{-4} to 9 · 10^{-4} m s^{-1}.

The VaCNT membrane allows a water flow velocity of water similar to the flow velocities in UF and NF membranes, while the thickness of the separation layer is 2−3 order of magnitudes larger than that of UF/NF membranes. This implies a low resistance of the VaCNT membrane.

2.2. Pure water permeabilities, slip lengths and enhancement factors

To determine how water transport was facilitated in the filtration with the VaCNT membranes, the pure water permeability, slip length and enhancement factor (EF, experimental permeability divided by Hagen−Poiseuille theoretical permeability) are shown in Supplementary Figure 2.

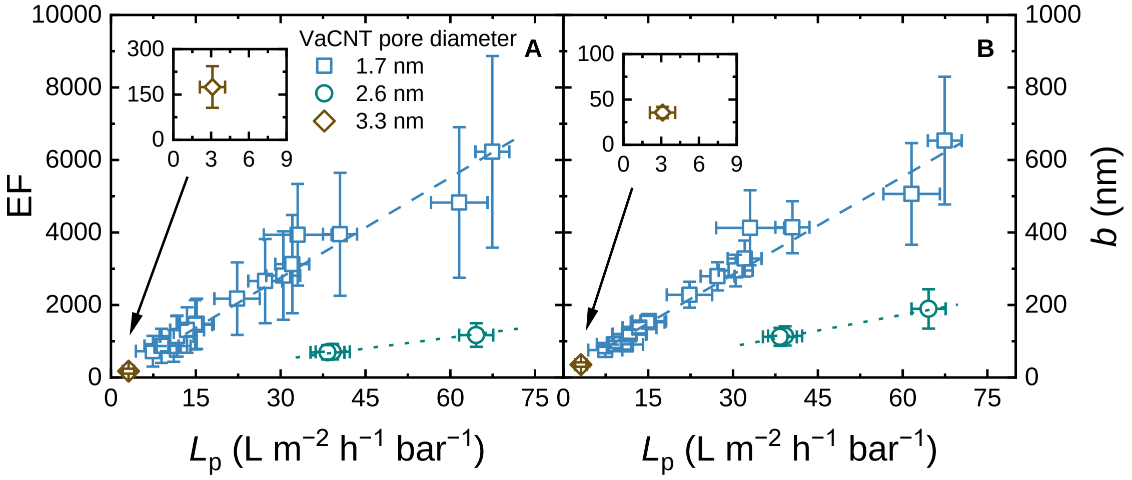

Supplementary Figure 2. Calculated enhancement factor (EF) and slip length b (B) as functions of experimentally-determined pure water permeability L_p, aggregated from 16, 3 and 1 data of VaCNT membranes with average pore diameters 1.7, 2.6 and 3.3 nm, respectively. Error bars represent propagated error from operational parameter variations.

The permeabilities of VaCNT membranes with pore diameter d_p of 1.7 nm are 9−67 L m^{-2} h^{-1} bar^{-1}, which is in the approximate range of NF [26, 39] and dense UF [40] membranes. The EFs and slip lengths of VaCNT membranes with d_p = 1.7 nm are 700−6000 and 75−655 nm, respectively (similar to the ranges reported by Holt *et al.* [41]).

The permeabilities of VaCNT membranes with d_p = 2.6 nm were similar to those of 1.7 nm diameter membranes, although both the EF and slip length of the 2.6 nm diameter ones were lower. The membrane with d_p = 3.3 nm has lower permeability (3 L m^{-2} h^{-1} bar^{-1}), slip length (30 nm) and EF (150) than those of the membranes with smaller pore diameters.

In all permeability tests, the slip length is very high (*i.e.* $b \gg \frac{d_p}{2}$) and EF is 2−4 orders of magnitude. Because of that, a plug-like flow condition can be assumed. The perfect plug flow is hypothetical and correspond to $b = \infty$ and hence EF = ∞. [21] The assumption of plug-like flow condition is applied for water only and may not be valid for other solutes such as SHs.

The exceptional water transport in VaCNT membranes compared with those of UF and NF (NF270) is also demonstrated *via* the thickness-independent expression of water permeability $L_p \cdot L$ (Supplementary Table 3). The values of $L_p \cdot L$ attained with VaCNT membranes are much higher than those attained with UF and NF membranes.

Supplementary Table 3. Comparison of water permeability between VaCNT membranes (three pore diameters) and UF/NF membranes.

**Membranes**	**VaCNT 1.7 nm**	**VaCNT 2.6 nm**	**VaCNT 3.3 nm**	**UF membranes**	**NF270**
Pore diameter (nm)	1.7 ± 0.7	2.6 ± 0.7	3.3 ± 0.8	1.6−5.4	0.8 ± 0.1 [a, 28]
Membrane / active layer thickness (μm)	45−56	26 ± 2	69 ± 1	0.5−1 [29]	0.02−0.04 [30, 31]
Water permeability L_P (L m^{-2} h^{-1} bar^{-1})	9−67	38−65	3.1 ± 1.0	4−88	13 ± 3
Thickness-independent expression of water permeability $L_p \cdot L$ (L μm m^{-2} h^{-1} bar^{-1})	500−2770	996−1680	215 ± 72	6−125	0.4 ± 0.2
Slip length (nm)	75−650	112−190	36 ± 6	-	-
Enhancement factor (EF)	720−6200	690−1200	174 ± 70	-	-

[a] Calculated based on the steric hindrance pore model described by Deen [37]

2.3. Surface hydrophilicity of the VaCNT membrane

Surface hydrophilicity facilitates water entry in the VaCNT membrane pores. The surface contact angle was measured with the captive bubble and sessile drop methods. The lower the contact angle, the more hydrophilic the membrane surface, and water may better penetrate the CNT pores. Photographs of the air bubble and water drop at the VaCNT membrane surface after <1, 10, 30 and 60 s are given in Supplementary Figure 3.

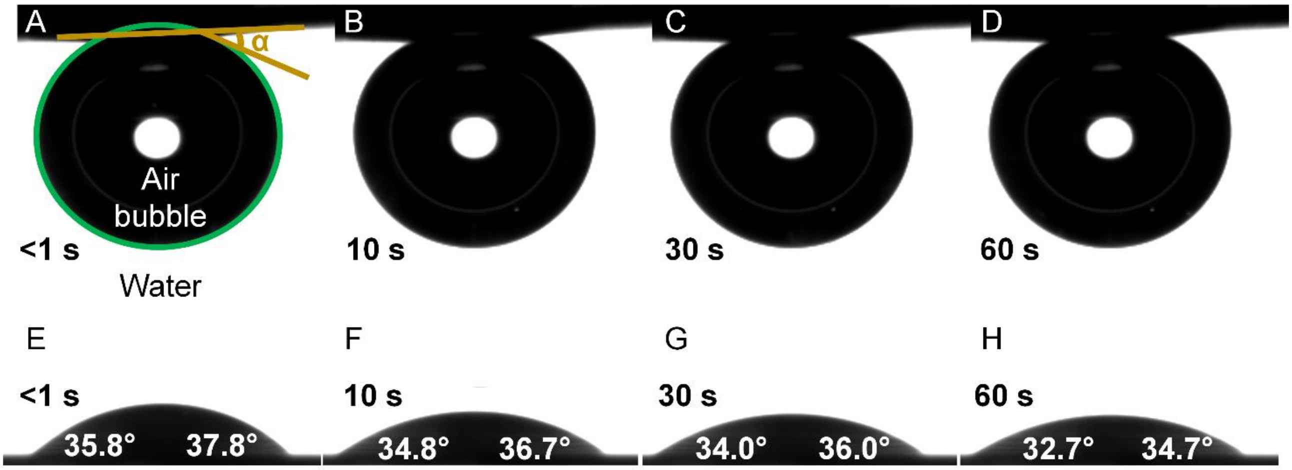

Supplementary Figure 3. A−D: an air bubble at the VaCNT membrane surface (captive bubble method) after <1, 10, 30 and 60 s since its release. The contact angle α is annotated in A. E−F: a water drop on the VaCNT membrane surface (sessile drop method) after <1, 10, 30 and 60 s since its release.

The contact angle in the captive bubble method was small (29.8° ± 0.3°), implying that the membrane surface is hydrophilic because of the strong capillary effect that saturates the membrane pores with water [42, 43] and functionalisation of carboxylate groups at the tip of the CNTs. [27] The size of the air bubbles and the contact angle did not change with time. With the sessile drop method, the contact angle decreased over time from 36° to 33° in 60 s due to the capillary effect caused by the pores, [42, 43] and water spreading on a non-flat surface (this surface can be observed in Figure 4 of the main text). It is then confirmed that the VaCNT membrane surface is hydrophilic and has moderately good wettability.

Supplementary Discussion 3. Experimental results

3.1. Hormone adsorption by the MF membranes

E2 breakthrough and adsorbed mass with the MF support membranes at varying fluxes are given in Supplementary Figure 4. The adsorbed mass of E2 by the VaCNT membranes can be determined by subtracting the VaCNT−MF membrane data from the MF membrane data. System adsorption (without dampener) is shown with the black diamond symbol.

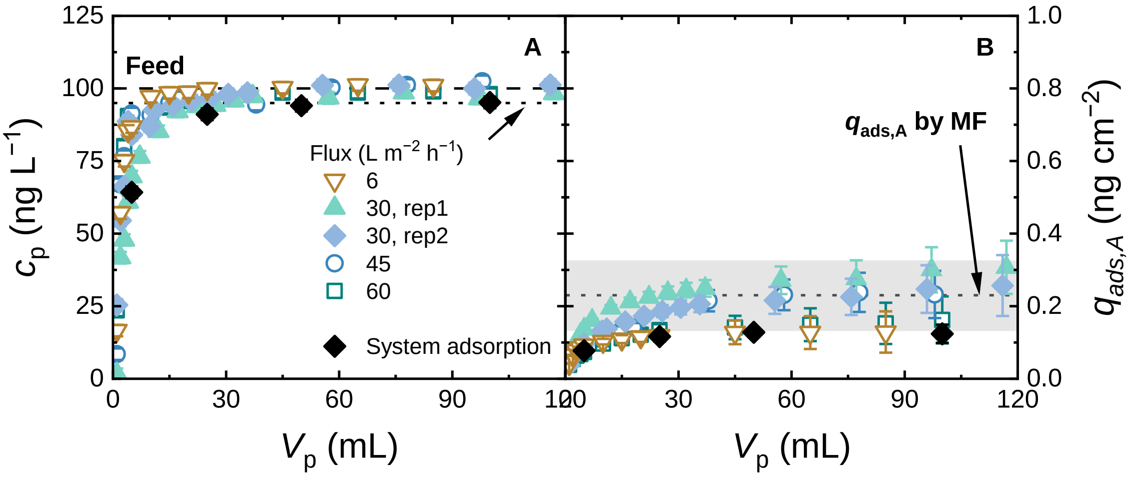

Supplementary Figure 4. Permeate E2 concentration c_p (A) and specific adsorbed mass (B) of MF support membranes vs. permeate volume at different fluxes. 100 ng/L E2, 1 mM $NaHCO_3$, 10 mM NaCl, pH 8.1 ± 0.2, 23.0 ± 0.2 °C. Adsorbed mass by the filtration system (without dampener) was 0.15 ng cm^{-2} (the black arrow in A indicate the feed concentration in this test, of 95 ng L^{-1}). Error bars represent propagated error from operational parameter variations and analytical error.

Complete breakthrough was achieved with all fluxes where the MF membrane material was saturated with SH, and adsorption was no longer significant [44] (in this case, where the permeate concentration reached the feed concentration). The adsorption saturation was approached at around 0.22 ng cm^{-2} where the permeate volume reached 45 mL in all experiments.

3.2. Removal of electrical conductivity

The electrical conductivities (ECs) of the feed and permeate were measured with in-line sensors to determine if there was any significant removal of salt by VaCNT membranes (Supplementary Figure 5). In the early experiments (30 L m^{-2} h^{-1} repeat 1, 45 L m^{-2} h^{-1}, and 6 L m^{-2} h^{-1} fluxes), some bubbles were stuck in the contactless permeate EC sensor and caused erroneous data. The tubing was later adjusted to ensure no bubbles accumulated. The EC of collected permeate samples from 5 mL were then determined with an external EC sensor (described in the Methods).

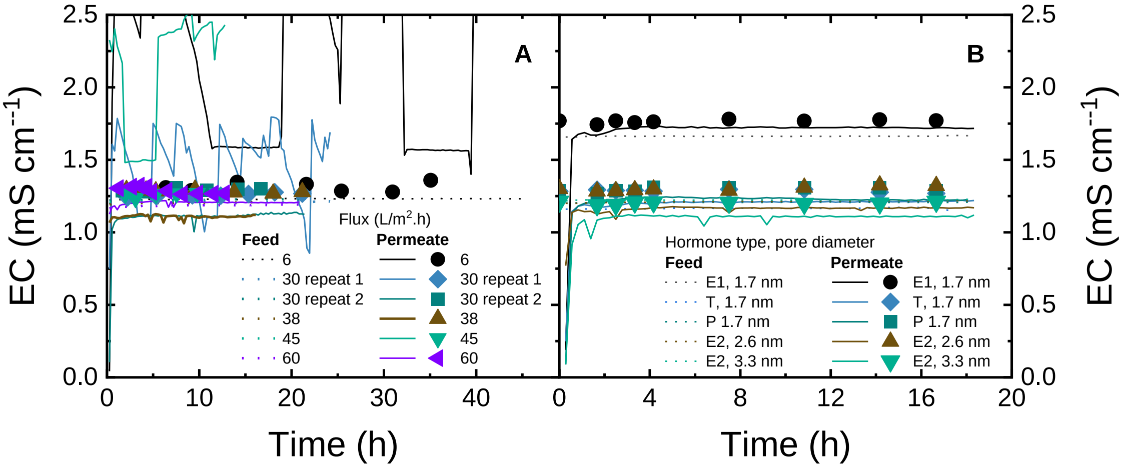

Supplementary Figure 5. Electrical conductivity (EC) in experiments with the VaCNT membranes measured with different sensors: in-line sensor for the feed (dotted line), in-line sensor for the permeate (solid lines), and offline sensor for the permeate samples (scatters).

The EC of the feed and permeate samples are the same, which implies that there was no significant removal of background electrolytes (1 mM $NaHCO_3$ and 10 mM NaCl).

3.3. Removal of ethanol

To investigate whether ethanol retention and hence concentration polarization of ethanol was significant, ethanol in the feed and permeate samples were quantified with the TOC analyser (Supplementary Figure 6).

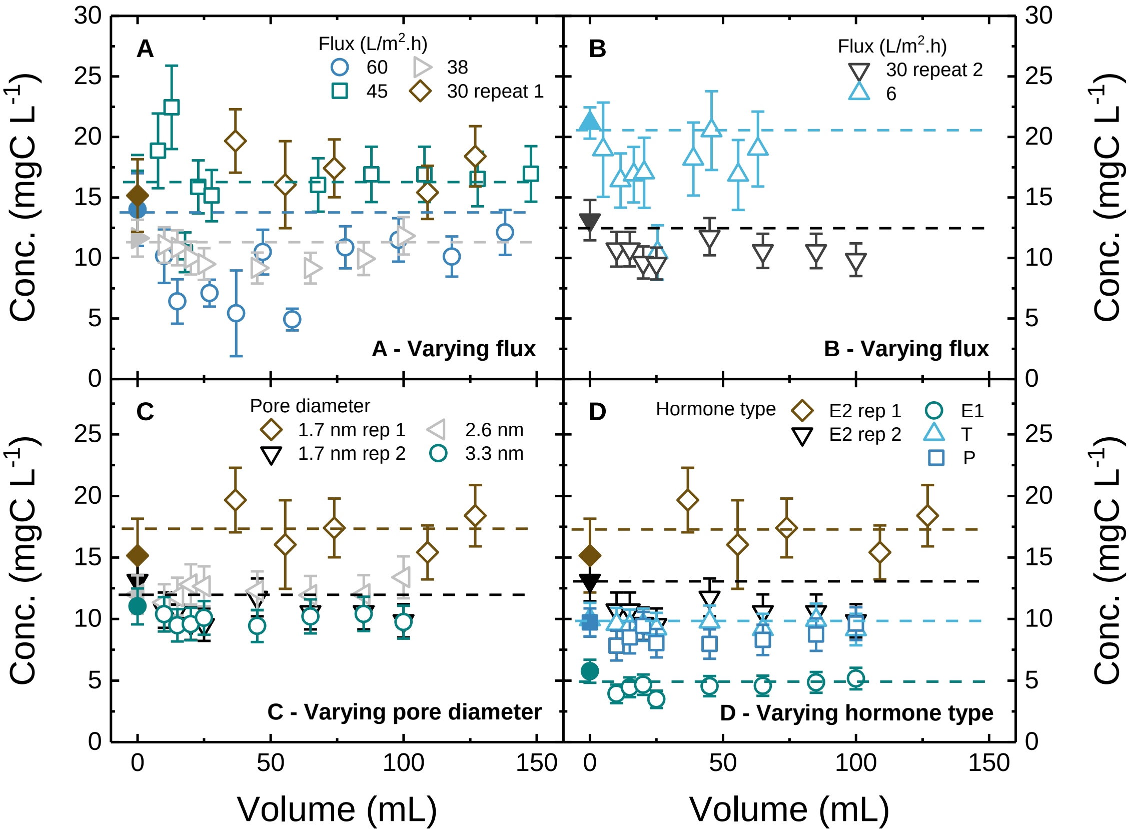

Supplementary Figure 6. Total organic carbon (TOC) concentrations measured with a TOC analyzer from all filtration experiments with VaCNT−MF membranes, with varying flux (A and B), pore diameter (C) and SH type (D). Filled points indicate the feed concentrations. Error bars represent propagated error from operational parameter variations and analytical error.

Insignificant retention of ethanol by VaCNT−MF membranes was observed, and the concentrations of ethanol in experiments with VaCNT membranes fall between 10 and 20 mgC L^{-1}.

3.4. Breakthrough of various hormones with 1.7 nm pore diameter VaCNT membranes

To verify whether a trend in adsorption can be identified with adhesive force, the breakthrough curves and adsorbed masses of the four SHs were compared in Supplementary Figure 7.

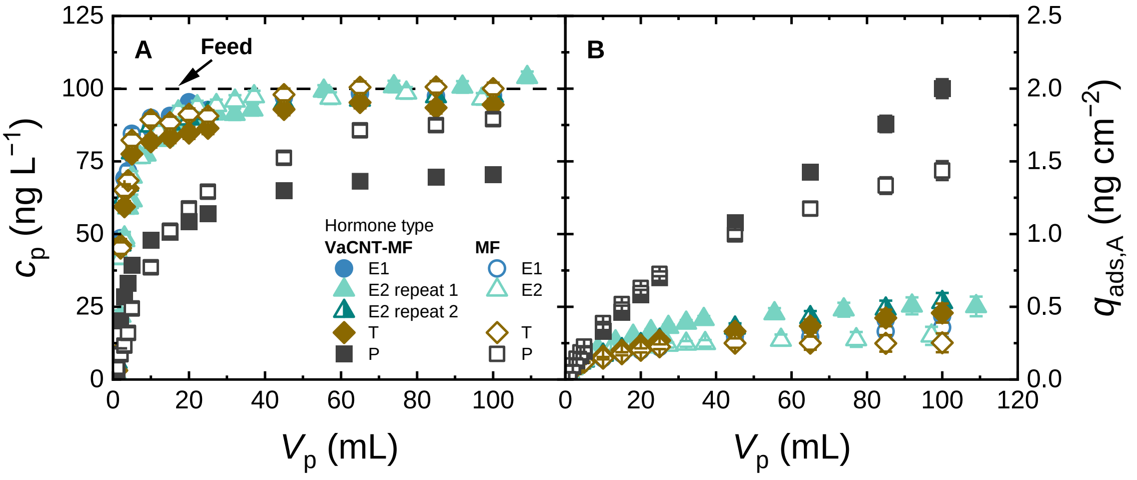

Supplementary Figure 7. Permeate concentration c_p (A) and specific adsorbed mass $q_{ads,A}$ (B) vs. permeate volume V_p with different SHs. Flux 27 ± 3 L m^{-2} h^{-1}, 100 ng L^{-1} E2, 1 mM $NaHCO_3$, 10 mM NaCl, pH 8.1 ± 0.2, 23.0 ± 0.2 °C. Error bars represent propagated error from operational parameter variations and analytical error.

No retention of E1, E2 and T was observed. For P, permeate concentration appeared to be level at 70 ng L^{-1} but this may be because of adsorption instead of retention. P (0.86 nm in diameter) is larger than E1, E2, and T (0.79−0.82 nm) but smaller than the VaCNT membrane pore diameter (~1.7 nm).

The adsorption of E1, E2 and T by VaCNT−MF membranes approached saturation after 100 mL. The adsorption of P by both VaCNT−MF and MF membranes did not reach saturation. The adsorbed mass of P by both MF and VACNT−MF membranes was higher than those of E1, E2 and T.

In conclusion, SH adsorption by the VaCNT membranes is specific to the SH type.

3.5. Adsorption affinity of VaCNT membrane for steroid hormones

Static adsorption with the VaCNT membrane was performed where convection through the pores is irrelevant, hence adsorption was dictated by the surface affinity for the SH (E2). The adsorption performance of the VaCNT membrane was benchmarked against other carbon-based nanoparticles to compare the surface accessibility. The relative adsorption affinities of the external and internal CNT surfaces were determined with the comparison between VaCNT membrane and single-walled carbon nanotube (SWCNT).

Data of carbon-based nanoparticles (MW-/SWCNT, graphene and fullerene C_{60} at the same mass of 2.5 mg) were taken from Nguyen *et al.* [45] (Supplementary Figure 8).

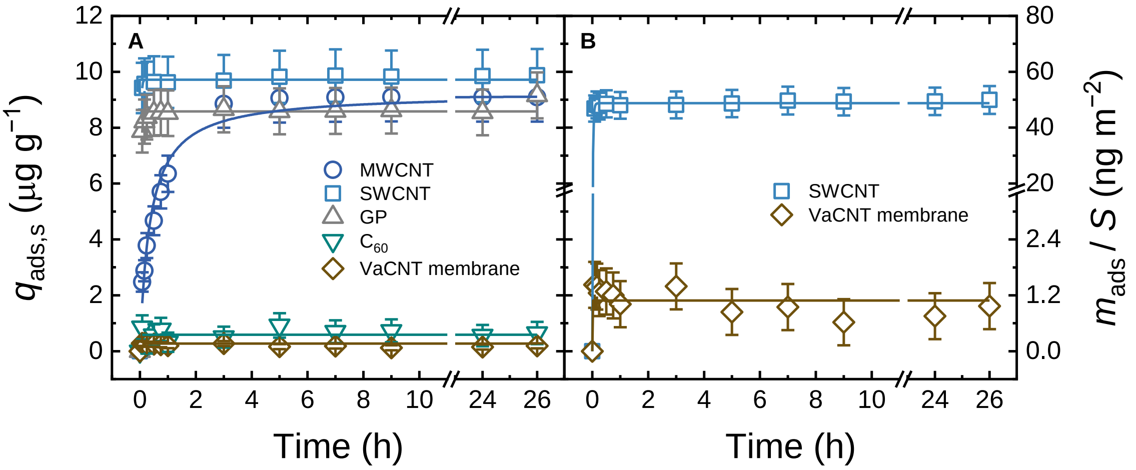

Supplementary Figure 8. Specific E2 adsorbed mass ($q_{ads,s}$) vs. time for different carbon-based nanoparticles and VaCNT membrane with the same mass of 2.5 mg (A), and E2 adsorbed mass per adsorbent surface area ($m_{ads,s}/\,S$) between SWCNT and VaCNT membrane (B). Data of polymer-based spherical activated carbon, multi-/single-walled carbon nanotube (MW-/SWCNT), graphene (GP), and C_{60} are taken from Nguyen *et al.* [45] 100 ng L^{-1} E2, adsorbent, 1 mM $NaHCO_3$, 10 mM NaCl, 20.0 ± 0.5 °C, pH 8.1 ± 0.2. Error bars represent propagated error from operational parameter variations and analytical error.

SWCNT and graphene displayed fast and high adsorption because their surfaces are accessible, whereas the adsorption by MWCNT suggests surface availability but lower accessibility. Adsorption by C_{60} was low because C_{60} formed strong aggregates that prevent surface access. [45] The specific adsorbed mass by VaCNT membrane was very low (around 0.22 µg g^{-1}, or 0.45 ng per cm^2 of filtration area, slightly higher than the filtration result at 6 L m^{-2} h^{-1} flux) which may imply the weak adhesive interaction. This adsorbed mass was even lower than that of an NF membrane (NF270), [46] of around 0.7 ng cm^{-2}. Weak adsorption at the internal surface is confirmed as the adsorbed mass per surface area of the VaCNT membrane was 30 times lower than that of SWCNT.

In conclusion, E2 adsorption was more favoured on the external surface, which could be attributed to the low electronic density at the internal surface [47, 48] that inhibits the non-covalent interactions (namely van der Waals interaction [49]) with E2.

3.6. E2 breakthrough with varying VaCNT membrane pore diameter

To determine whether the VaCNT membrane pore diameter in the nanometre range limited adsorption, the E2 breakthrough curves and adsorbed masses with VaCNT membranes that have three pore diameters (1.7, 2.6 and 3.3 nm) were compared in Supplementary Figure 9.

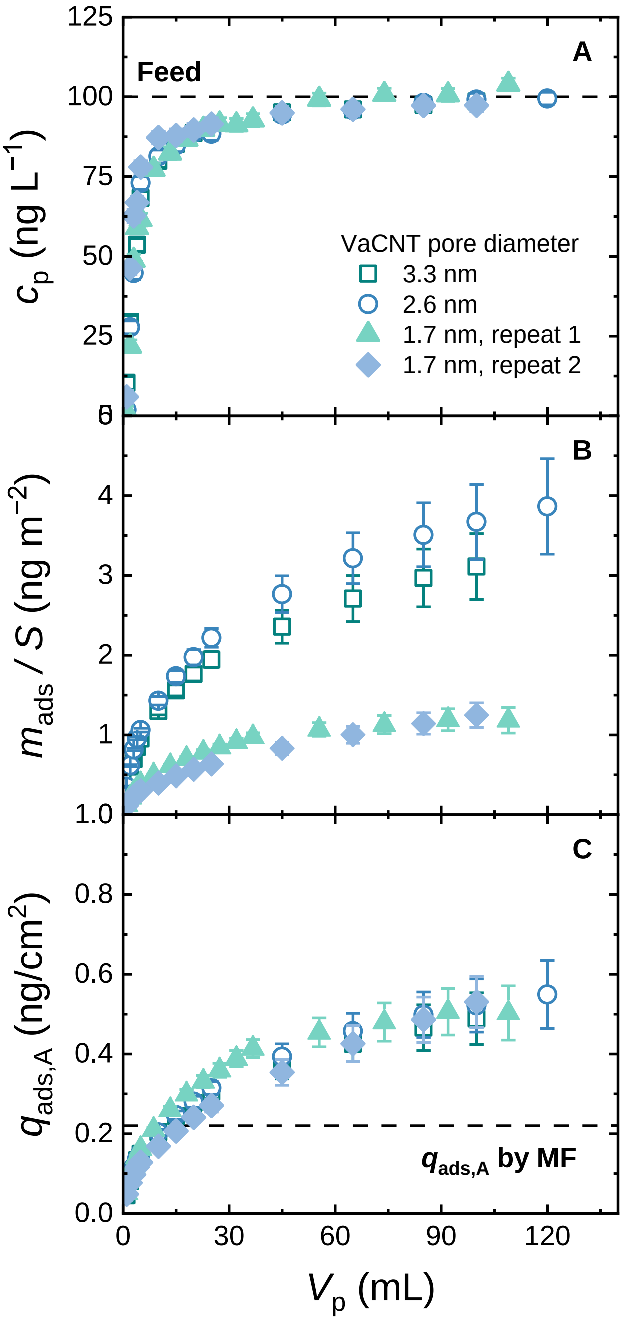

Supplementary Figure 9. Permeate E2 concentration c_p (A), adsorbed mass per CNT surface area (B) and specific adsorbed mass (C) of VaCNT−MF membranes with varying VaCNT pore diameter. 100 ng L^{-1} E2, 1 mM $NaHCO_3$, 10 mM NaCl, pH 8.1 ± 0.2, 23.0 ± 0.2 °C. Error bars represent propagated error from operational parameter variations and analytical error.

Complete breakthrough was observed with all pore diameters. Adsorption saturation (where the adsorbed mass no longer increases) was approached at around 100 mL. No trend in the specific adsorbed mass with pore diameter can be determined.

From the trend in adsorbed mass per CNT surface area, it appears that the CNT surface did not limit adsorption. The trend in adsorption kinetics could not be determined because the data points for different membrane pore diameters overlap.

3.7. Estimation of the diffusion time in the membrane pores

The pore diffusivity of E2 in a VaCNT membrane (with a pore diameter 1.7 nm) was determined. The feed and permeate E2 concentrations and the adsorbed mass of E2 are given in Supplementary Figure 10. At the quasi-steady state/adsorption equilibrium, SH was no longer significantly adsorbed, the diffusivity was determined from equation (S6) in Supplementary Table 6. The diffusivity measurement was not corrected with the actual number of conducting CNTs, while the diffusion hindrance by the membrane boundary layer and pore entrance had not been accounted.

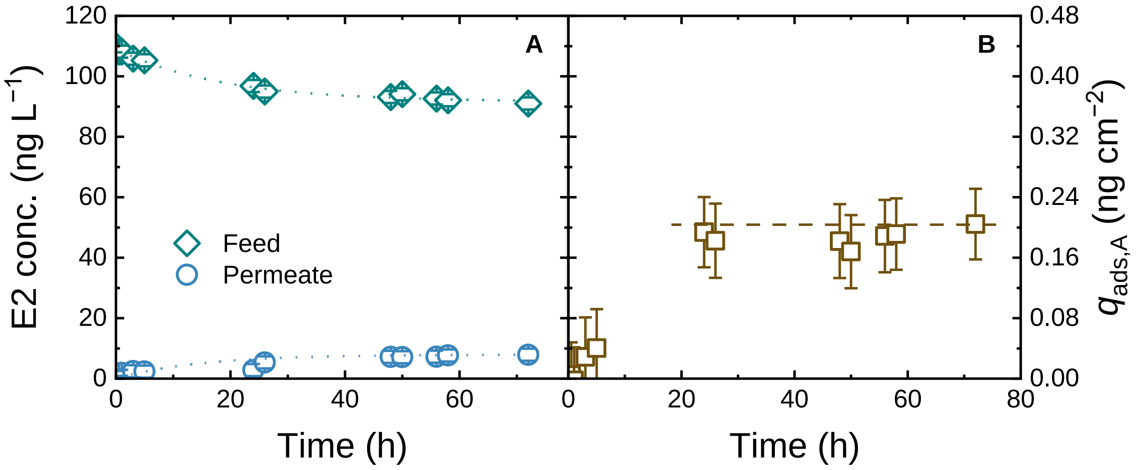

Supplementary Figure 10. Feed and permeate E2 concentrations (A), and adsorbed mass (B) vs. time in diffusion cell experiment. The initial feed solution contained 105 ng L^{-1} E2, 1 mM $NaHCO_3$ and 10 mM NaCl, 23.5 ± 0.5 °C, pH 8. Error bars represent propagated error from operational parameter variations and analytical error.

The adsorbed mass at the steady state, or adsorption equilibrium (0.20 ± 0.05 ng) was reached in 7 to 24 h. The concentration gradient between the feed and permeate sides was 80 ng L^{-1} after 75 h of diffusion.

The measured diffusivity of E2 is 1.0 · 10^{-10} m^2 s^{-1} from equation (S6) is lower but in the same order of magnitude as the bulk diffusivity (5.6 · 10^{-10} m^2 s^{-1}), [36] and similar to the hindered diffusivity (1.2 · 10^{-10} m^2 s^{-1}). [37] The measured diffusivity ia probably underestimated because this value is not only the result of pore diffusion, but also the hindrance from membrane boundary layer and the pore entrance effect.

The (radial) diffusion time in the membrane pores allows the determination of whether the SH reaches the membrane surface and allows adsorption to occur. This radial diffusion time from the pore centre to the pore wall is estimated *via* the follow equation. [50]

$$t_D = \frac{x^2}{D} \qquad \text{(S2)}$$

where D (m^2 s^{-1}) is the SH diffusivity in the pore and x (between 0 and $\frac{1}{2}d_p$) is the diffusion distance in the radial direction. If the diffusion time is larger than the hydraulic residence time t_R, the SH molecule is not adsorbed to the pore wall. The orders of magnitude of the maximum t_D (defined when

$x = \frac{1}{2} d_p$) and t_R for the VaCNT, UF and MF membranes are given in Supplementary Table 4. For NF membranes, the pore diameter is close to the SH diameter, so the diffusion time is negligible.

Supplementary Table 4. Membrane properties, hydraulic residence time and radial diffusion time summarised for the VaCNT, NF, UF and MF membranes.

Parameter	Units	NF	VaCNT	UF	MF
Pore diameter	nm	1 [28]	2	20	200 [51]
Separation layer thickness	µm	0.03 [30, 31]	50	0.5 [29]	100 [51]
Porosity	%	3 [12, 13]	3	8 [25]	70 [51]
Modelled flux	$L\ m^{-2}\ h^{-1}$	100	100	1000	1000
Hydraulic residence time [a]	s	10^{-5}	0.1	0.1	0.1
Radial diffusion time [a, b, c]	s	Instantaneous [d]	10^{-11}	10^{-7}	10^{-5}
Adsorption	-	High	Zero	Low	Low

[a] Only the order-of-magnitude estimation is given.
[b] Diffusion is from the pore centre to the wall. The contribution of the van der Waals interaction to the radial movement of SH is neglected.
[c] In UF and MF membrane pores, the bulk diffusivity of SH in the pores of $5.6 \cdot 10^{-10}\ m^2\ s^{-1}$ (calculated with the equation proposed by Worch [36]) is used to calculate the diffusion time.
[d] Negligible radial diffusion distances because the pore diameter is approximately the diameter of SH (0.8 nm).

The diffusion time in VaCNT membranes was very short, indicating that the SH reached the pore wall as soon as it entered the membrane pore. The diffusion times in UF and MF membranes were longer than that in the VaCNT membrane, but are still several orders of magnitude shorter than the hydraulic residence time. This means the SH molecules could reach the pore wall during filtration, and adsorption of these molecules was relevant.

Supplementary Methods

Supplementary Discussion 4. Materials

4.1. Properties of steroid hormones

The properties of the four steroid hormones (SHs) are given in Supplementary Table 5. The lower solubility values in water than those in ethanol/methanol imply the capability of SH to partition towards ethanol in an ethanol/water mixture and form clusters. Since ethanol can interact more strongly with the carbon nanotube (CNT) wall than water, [52, 53] ethanol and the formation of hormone−ethanol clusters can affect the hormone−wall interaction.

Supplementary Table 5. Molecular structures, molecular weights, hydrodynamic diameters and solubilities in water and alcohols (ethanol and methanol) of four SH types. N.D.: not determined.

**Parameter**	**E1**	**E2**	**T**	**P**
Chemical formula	$C_{18}H_{22}O_2$	$C_{18}H_{24}O_2$	$C_{19}H_{28}O_2$	$C_{21}H_{30}O_2$
Molecular structure	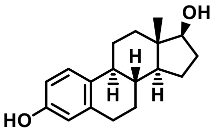	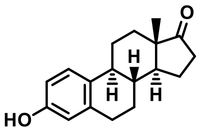	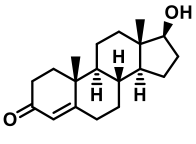	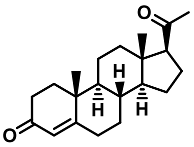
Molecular dimension (nm · nm · nm)*	0.35 · 0.48 · 1.05	0.36 · 0.48 · 1.05	0.35 · 0.55 · 1.12	0.35 · 0.55 · 1.19
Molecular weight (Da)	270	272	288	314
Stokes diameter (nm) [36]	0.79	0.80	0.82	0.86
Solubility in water at 25°C (mg L^{-1})	0.80–1.3 [54-56]	0.16–5.0 [54, 56-59]	20–48 [57, 59-63]	7.9–17 [57, 59, 60, 64, 65]
Solubility in methanol at 30°C (g L^{-1})	4.5–5.4 [66, 67]	35.3 [68]	224 [69]	N.D.
Solubility in ethanol (g L^{-1})	5.4–7.1 (30°C) [66, 67]	37–42 (30°C) [68]	N.D.	54 (21°C) [70]

* Estimated from Chem3D (ChemOffice, PerkinElmer, USA) models. [71]

4.2. Summary of vertically aligned carbon nanotube (VaCNT) membrane fabrication

The fabrication of vertically aligned carbon nanotube (VaCNT) membranes is schematically described in Supplementary Figure 11 and includes the following steps: 1) deposition of catalyst nanoparticles (Fe/Mo or Fe only) on a silicon support by thermal evaporation, 2) chemical vapour deposition growth of aligned CNTs, 3) chemical vapour deposition of parylene-N as barrier material, and 4) plasma etching to open the CNT pores and introduce oxygen containing groups at the tips.

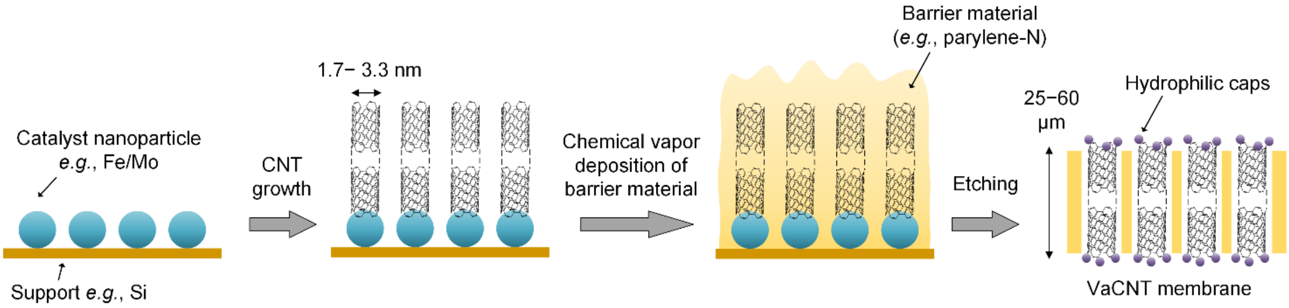

Supplementary Figure 11. Schematic of the VaCNT membrane fabrication steps, adapted from Bui *et al.* [72]

4.3. Estimation of VaCNT pore diameter

The average size of nanotubes in the single-walled carbon nanotube (SWCNT) ‘forest’ after the CNT growth step was determined from a rich dataset of transmission electron microscopy (TEM) images (for example, Supplementary Figure 12 A) with >200 measurements as described by Bui *et al.* [72] From this value, the width (diameter) of the VaCNT membrane inner pores where water and solutes accommodate was then determined.

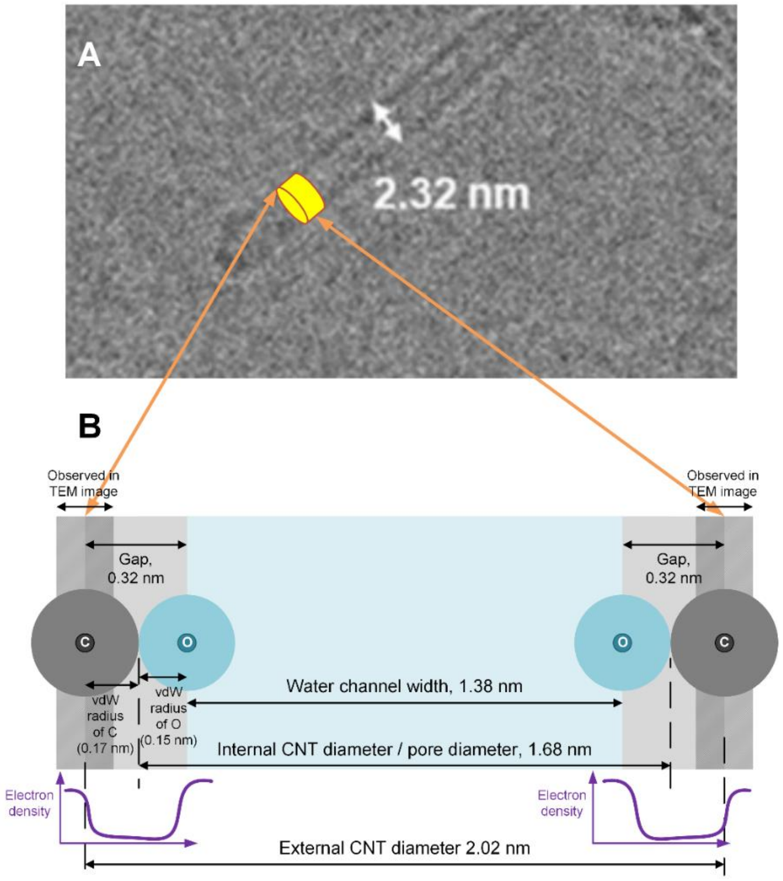

Supplementary Figure 12. A – TEM image of an SWCNT in the forest taken from Buchsbaum *et al.*, [73] showing a CNT diameter of 2.32 nm. B – Schematic of the cross-section along the axial direction of a CNT with a measured diameter of 2.02 nm, in which the van der Waals radii of carbon and oxygen atoms, and the internal CNT diameter (also the VaCNT pore diameter), are illustrated.

The CNT size obtained from TEM images is roughly the distance between the two carbon atoms at their atomic centres. A 0.32 nm (vacuum) gap where the electron density is minimal (Supplementary Figure 12 B) is determined as the sum of the van der Waals radii of the carbon (0.17 nm) and oxygen atoms (0.15 nm). [74] When two atoms are at a shorter distance than the van der Waals distance, they will repel each other to reduce the interaction potential energy. [15]

The VaCNT membrane inner pore diameter is the average CNT size determined from TEM imaging minus twice the van der Waals radius of CNT’s carbon (0.34 nm). [75]

Supplementary Discussion 5. Analytical techniques

5.1. Calibration of liquid scintillation counter for steroid hormone quantification

SH calibration curves are given in Supplementary Figure 13 A. Supplementary Figure 13 B shows the calibration curves with low-concentration data to estimate the detection limit (LOD). The standard solutions (concentrations 0.2, 1, 10, 50 and 100 ng L^{-1}) were prepared directly by dilution from the fresh stock solutions with a concentration 10 µg L^{-1}.

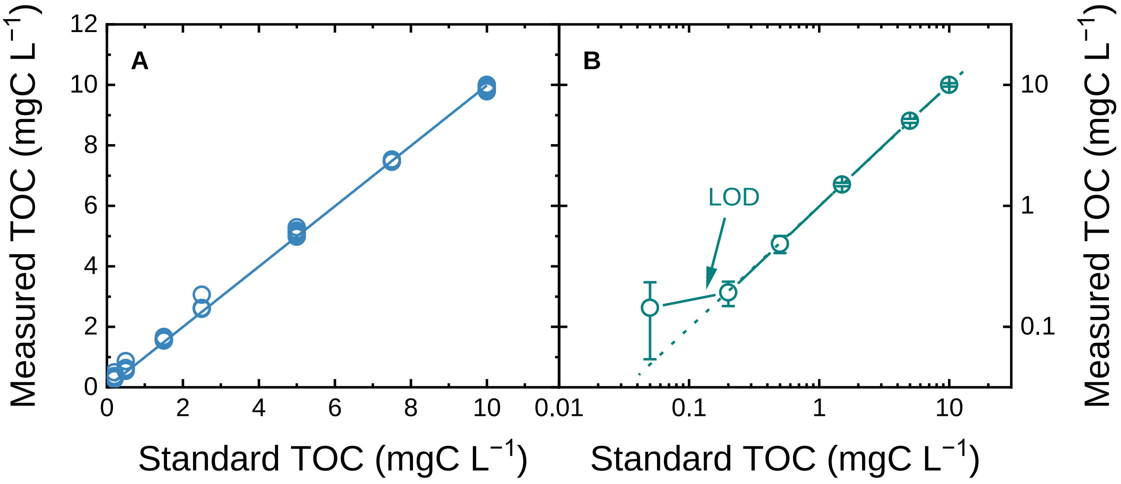

Supplementary Figure 13. A – Multiple calibration results (activity vs. standard SH concentration) in this study. B – Average activity vs. expected SH concentration with another E2 batch, determined from >40 measurements for each concentration; the error bar indicates the standard deviation between calibrations.

A linear relationship was found between activity and standard concentration. A little deviation (below 2% for concentrations 10−100 ng L^{-1} and 10% for the low concentration 1 ng L^{-1}) was obtained between calibration repeats for E2, which indicates the high accuracy of LSC. The LOD is estimated by inspecting a break in the calibration regression [76] to be around 0.1−0.2 ng L^{-1}.

5.2. Calibration of the total organic content analyser for ethanol quantification

Total organic carbon (TOC) calibration in the range of 0.2−10 mgC L^{-1} with potassium hydrogen phthalate (dissolved in Milli-Q water from powder, 99.5%, Merck Millipore) is shown in Supplementary Figure 14.

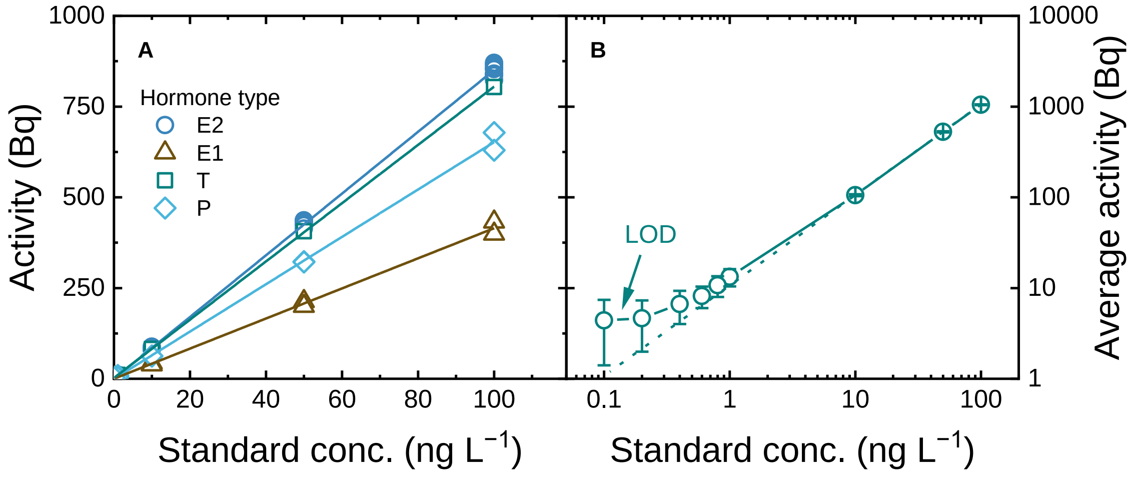

Supplementary Figure 14. Total organic carbon (TOC) calibration with three data sets, presented as signal reported by the TOC analyzer vs. expected concentration. The error range indicates the maximum variation between calibrations.

A linear relationship was found between the TOC signal and expected concentration in the range of 0.5–10 mgC L^{-1}. The deviation between calibration repeats (4% for 1.5–10 mgC L^{-1}, 16% for 0.5 mgC L^{-1} and 25% for 0.2 mgC L^{-1} concentrations) was large compared with the LSC method with its respective measurement range. The LOD estimated *via* inspecting a break in the calibration regression [76] is 0.2 mgC L^{-1}.

Supplementary Discussion 6. Calculations

6.1. Formulae to calculate experimental parameters

Supplementary Table 6. List of formulae applied in static adsorption, diffusion, and filtration experiments.

Eq. No.	Parameter	Units	Formula	Ref.
**Static adsorption**				
(S3)	Adsorbed mass at time t ($m_{ads(t)}$)	ng	$m_{ads(t)} = (c_f - c_{(t)}).V_f$	
(S4)	Specific adsorbed mass ($q_{ads,s(t)}$)	ng g^{-1}	$q_{ads,s(t)} = \dfrac{m_{ads(t)}}{\text{mass of VaCNT membrane}}$	
**Diffusion**				
(S5)	Adsorbed mass at time t ($m_{ads(t)}$)	ng	$m_{ads(t)} = c_{f(t)}\, V_{f(t)} - c_{p(t)} V_{p(t)}$	
(S6)	Diffusivity (D) of SH in VaCNT membrane pore*	$m^2\ s^{-1}$	$D = -\dfrac{L \cdot V}{2 \cdot \varepsilon \cdot A_m \cdot t} \cdot \ln \dfrac{c_{f(t)} - c_{p(t)}}{c_{f0} - c_{p0}}$	77
**Filtration**				
(S7)	VaCNT membrane porosity (ε)	-	$\varepsilon = \pi \left(\dfrac{d_p}{2}\right)^2 \dfrac{N_{\mathrm{CNT}}}{A_{\mathrm{m}}}$	72

(S8)	Tortuosity (α)	-	$\alpha = \frac{\text{Length of actual path}}{\text{Length of hypotherical straight path}}$	78
(S9)	Water flux (J_{exp})	L m^{-2} h^{-1}	$J_{exp} = \frac{dV}{A_{\mathrm{m}}\, dt} = \frac{dm}{\rho\, A_m\, dt}$	78
(S10)	Membrane permeability ($L_{P,exp}$)	L m^{2} h^{-1} bar^{-1}	$L_{p,exp} = \frac{J_{exp}}{\Delta P}$	78
(S11)	Slip length (b)	nm	$b = \frac{L_{P,exp}\, \mu\, L}{16\pi\, \varepsilon\, d_p} - \frac{d_p}{8}$	41
(S12)	Theoretical permeability (Hagen−Poiseuille equation)	L m^{2} h^{-1} bar^{-1}	$L_{p,HP} = \frac{\varepsilon}{8\, \mu\, L} \left(\frac{d_p}{2}\right)^2$	38
(S13)	Enhancement factor (EF)	-	$\mathrm{EF} = \frac{L_{p,exp}}{L_{p,HP}} = 1 + \frac{8\, b}{d_p}$	41
(S14)	Average flow velocity in cylindrical pores (v_{water})	m s^{-1}	$v_{\mathrm{water}} = \frac{J_{exp}}{\varepsilon}$	78
(S15)	Hydraulic residence time in cylindrical pores (t_R)	s	$t_R = \frac{L}{v_{water}} = \frac{\varepsilon\, L}{J_{exp}}$	
(S16)	Péclet number (Pe)	-	$Pe = \frac{L\, v_{\mathrm{water}}}{D_{\mathrm{water}}}$	
(S17)	Adsorbed mass of E2 (m_{ads})	ng	$m_{ads} = m_f - \sum m_p = c_f \sum V_p - \sum (V_p\, c_p)$	
(S18)	Specific adsorbed mass of E2 ($q_{ads,A}$)	ng cm^{-2}	$q_{ads,A} = \frac{m_{ads}}{A_m}$	

where:

A_{m}= 2 cm^2 is the membrane area,

ρ (g L^{-1}) is the water density (ρ = 1000 ± 3 g L^{-1} at a temperature of 23.0 ± 0.2 °C [79])

$\frac{dV}{dt}$ (mL min^{-1}) and $\frac{dm}{dt}$ (g min^{-1}) are the changes in permeate volume and mass with time; m is measured with a balance

ΔP (bar) is the applied pressure, measured by subtracting the pressure measured with the permeate-side sensor from that with the feed-side sensor

ε is the VaCNT membrane porosity (see Table 1 of the main text),

N_{CNT} is the number of CNTs determined from the CNT growth during membrane fabrication

D_{water} is the diffusivity of water in the nanopore (a bulk diffusivity value of $2.3 \cdot 10^{-9}$ m^2 s^{-1} is assumed [80])

d_p (m) is the VaCNT membrane pore diameter (given in Table 1 of the main text)

μ (kg m^{-1} s^{-1}) is the viscosity of water ($\mu = 9.33 \cdot 10^{-4}$ kg m^{-1} s^{-1} at a temperature of 23 °C [79])

L (m) is the VaCNT membrane thickness (see Table 1 of the main text)

m_f (ng), V_f (L) and c_f (ng L^{-1}) are the E2 mass, solution volume and E2 concentration of the feed

m_p (ng), V_p (L) and c_p (ng L^{-1}) are the E2 mass, solution volume and E2 concentration of the permeate

$c_{(t)}$ (ng L^{-1}) is the E2 mass and concentration at time t in static adsorption

*Quasi-steady-state in diffusion cell experiment assumes that the flux of the solute leaving the feed compartment is equal to the flux of the solute entering the permeate compartment. Another assumption is that the difference in liquid volume between the feed and permeate is negligible because of very weak osmotic pressure. The actual diffusion, pore entrance effect and membrane boundary layer resistance may affect the determined diffusivity so the measurement cannot be accurate.

6.2. Calculations of VACNT surface area and adsorption capacity

The specific surface area (SSA) of the VaCNT membrane was estimated from the membrane pore diameter, porosity, and thickness. The theoretical adsorption capacity was then determined from the SSA. The assumptions included a uniform membrane pore diameter of 1.7 nm, membrane porosity of 3.4%, and membrane thickness of 50 µm (Supplementary Table 7).

Supplementary Table 7. Calculations to determine the porosity, internal surface area and E2 adsorption capacity for the 1.7 nm VaCNT membrane. Note: it is assumed that the capping percentage is 0% (all the nanotubes are uncapped). For instance, if the capping percentage increased to 50%, the porosity, internal surface area and E2 adsorption capacity would be reduced to half.

**Parameter (units)**	**Value**	**Comments**
VACNT membrane thickness L (µm)	50	Measured at LLNL (SEM cross-section and optical microscopy) [27]
VACNT average distance between the wall's centres (nm)	2.0	Measured at LLNL (TEM analysis of multiple nanotubes (>300) from the same batch) [27]
VaCNT average internal tube diameter, *i.e.* membrane pore size d_p (nm)	1.7	Distance between the wall's centres subtracted by the van der Waals diameter of carbon (0.34 nm)
Area of a single VaCNT pore cross-section A_p (m^2)	$2.2 \cdot 10^{-18}$	$A_p = \pi \left(\frac{d_p}{2}\right)^2$
Internal surface area of each VaCNT tube A_{int} (m^2)	$2.6 \cdot 10^{-13}$	$A_{\text{int}} = \pi\, d_p\, L$
No. of nanotubes per m^2 of VaCNT surface $\frac{N_{\text{CNT}}}{A_{\text{m}}}$ (m^{-2})	$1.5 \cdot 10^{16}$	Calculated from the experimental weight gain method considering the VaCNT tube length / diameter as described elsewhere [27]
VaCNT membrane porosity ε	0.034	See equation (S7). For comparison, the porosity of NF membranes is between 2% and 32% [7], and the porosity of UF membranes ranges between 1% and 20% [81-83]. MF membrane porosity can reach 70%. [51]
External surface area per m^2 of filtration (membrane) area K_{ext} (m^2 m^{-2})	0.97	$K_{\text{ext}} = 1 - A_p \frac{N_{\text{CNT}}}{A_{\text{m}}}$
Internal surface area per m^2 of filtration (membrane) area K_{int} (m^2 m^{-2})	4000	$K_{\text{int}} = A_{\text{int}} \frac{N_{\text{CNT}}}{A_{\text{m}}}$
Membrane density ρ_{m} (weight per filtration area) (g m^{-2})	22	$\rho_{\text{m}} = \frac{m_{\text{m}}}{A_{\text{m}}}$ where m_m is the mass of a membrane coupon. The ρ_{m} value was determined from m_{m} and A_{m} of 10.6 mg and 4.91 cm^2, respectively.
**Theoretical specific surface area (SSA) of VaCNT membrane S_{int} (m^2 g^{-1})**	**180**	$S_{\text{int}} = \frac{K_{\text{int}}}{\rho_{\text{m}}} = A_p \frac{N_{\text{CNT}}}{m_{\text{m}}}$
Cross-sectional area of each E2 molecule (nm^2)	0.5	Estimated from the hydrodynamic diameter of the SH d_{SH} (for example, d_{SH} = 0.8 nm for E2) assuming that the SH molecule is spherical. $A_{\text{SH}} = \pi \left(\frac{d_{\text{SH}}}{2}\right)^2$
Max no. of E2 molecules that can occupy the internal surface of 1 cm^2 of VaCNT membrane ρ_{E2} (cm^{-2})	$1.7 \cdot 10^{15}$	$\rho_{E2} = \frac{S_{\text{SH}}}{A_{\text{SH}}} \frac{A_{\text{m}}}{m_{\text{m}}} = \frac{S_{\text{SH}}}{A_{\text{SH}}\, \rho_{\text{m}}}$

Weight of each E2 molecule m_{E2} (ng)	$4.5 \cdot 10^{-13}$	$m_{E2} = \mathrm{MW}_{E2}\, N$<br>where MW_{E2} and N are the molecular weight of E2 (272 g mol^{-1}) and Avogadro constant ($6.022 \cdot 10^{23}$ mol^{-1}).
**Max E2 adsorption capacity per cm^2 of membrane $q_{max,A}$ (ng cm^{-2})**	**770**	$q_{max,A} = \rho_{E2}\, m_{E2}$

Supplementary Discussion 7. Filtration system characterisation

7.1. Membrane filtration protocol

The filtration protocol is adapted from Nguyen *et al.* [25] and described in Supplementary Table 8. The main modifications in this work are: filtration was done in dead-end mode (with the needle valve closed the pump flow rate is set to ≤ 0.2 mL min^{-1}), and more samples were collected at the start of filtration (in the first five samples, the volume collected in each sample was 1 mL).

Supplementary Table 8. Filtration protocol, adapted from Nguyen *et al.* [25]

No.	Step	Conditions	Justification
1	Membrane conditioning	The VaCNT membrane is submerged in Milli-Q water for 1 h and then carefully rinsed.<br>The MF membrane is submerged in 50/50 ethanol/water (v./v.) for 15 min, and then rinsed thoroughly with Milli-Q water.	To permeate the membrane and wash off additives
2	System flushing	Without the membrane, set the pump flow rate at 100 mL min^{-1} and flush the entire system with Milli-Q water for 3 min.	To prepare for a new experiment
3	System drying	Connect the outlet of purge valve PV (see Figure 10, main text) with the synthetic air line. Flush the system with synthetic air for 5 min.	To remove water residues, especially in the membrane cell outlet and switching valve.
4	Pure water permeability test (before SH filtration)	Mount the membranes (VaCNT membrane on top of an MF membrane) with a torque of 2 N m (using a torque wrench). The water chiller is set at 23 °C. Pump flow rate is set at 0.1 mL min^{-1} (equivalent flux is 30 L m^{-2} h^{-1}). It then takes around 30−60 min until the permeate flux is stable. Filtration continues for at least 15 min at constant permeate flux to determine the permeability.	To determine the pure water permeability of the membrane and make sure the membrane has no defects (if the membrane is broken or has pinholes, the pressure will be below 0.2 bar).
5	Filtration test with SHs	Feed volume is 250 mL. Disconnect the inlet to the membrane cell and replace the water inside the tubing with feed solution (this takes 1 min with the pump flow rate set to 50 mL min^{-1}).<br>Turn off the pump and reconnect the inlet to the membrane cell. The water chiller is set at 23 °C. Operate the filtration at experimental fluxes*. Sample collection was based on permeate volumes:<br>Samples 1−5: 1 mL each<br>Samples 6−9: 5 mL each<br>Samples 10 onwards: 20 mL each	To investigate SH removal by VaCNT membranes.<br>The system's dead volume is 24.5 mL and the dead volume of the permeate tubing is 0.42 mL. Therefore, the purging with 50 mL of feed solution is justified.<br>The first and second permeate samples (each 1 mL) might contain residue water from the membrane cell.

		In all experiments, the filtration system was operated overnight.	
6	Pure water permeability test (after filtration)	Change the feed to Milli-Q then follow the same procedure described in step 4.	To determine the pure water permeability after the filtration
7	Membrane removal and storage	The membrane coupons are removed, dried in air for 1 h and stored in a plastic petri-dish.	
8	System flushing	The system is flushed with 1 M NaOH solutions for 15 min, then with warm deionised water (~40 °C) for 15 min, and finally with Milli-Q water for 15 min. The pump flow rate was set at 30 mL min^{-1} in all the steps.	NaOH and warm water flushing remove residual organic matter.

* In the experiment with the lowest flux (6–7 L m^{-2} h^{-1}, or 0.02 mL min^{-1}), the needle valve (NV in Figure 10, main text) was partially open, letting only ~30% of the feed solution to pass through the membrane. The other 60% was collected in a bottle that was connected to the NV outlet.

7.2. Adsorption by the filtration system (without membrane) with varied flow configurations

The adsorption of SHs to the filtration system (stainless steel and plastic tubes, pump, membrane cell and switching valve; without membranes) is a source of error and must be avoided as much as possible, especially when the membrane can adsorb very little SHs. Different configurations (dead-end vs. cross-flow, with or without a dampener) were evaluated for the filtration protocol via the adsorbed mass of E2 (Supplementary Figure 15).

In dead-end mode, the flux was fixed at 300 L m^{-2} h^{-1} and multiple permeate samples were collected in separate vials. In cross-flow mode with cross-flow velocities of 0.4–0.8 m s^{-1}, all the permeate and retentate are recycled into the feed bottle and the concentration in this bottle was determined at time intervals. The cross-flow data were obtained from Imbrogno and Schäfer. [26]

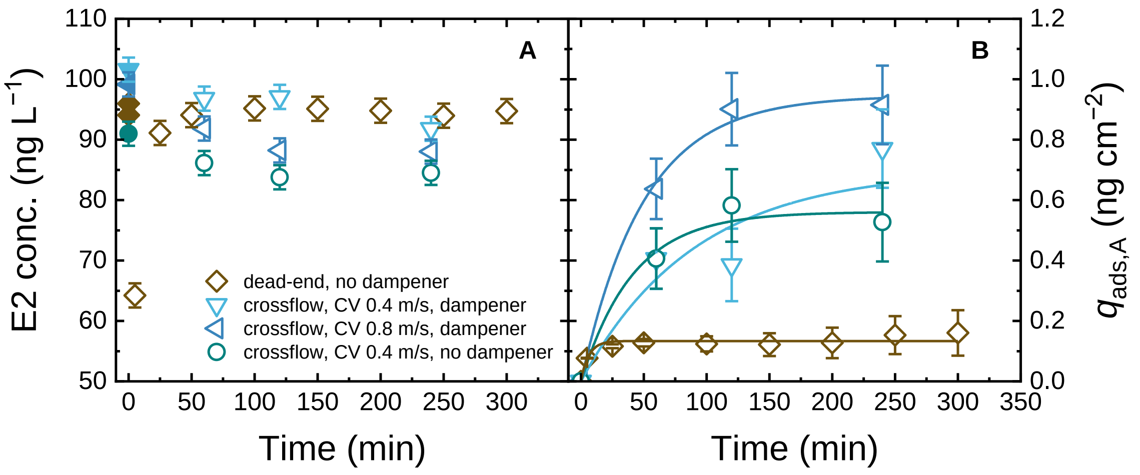

Supplementary Figure 15. E2 concentration (A) and adsorbed mass $q_{ads,A}$ (B) of the filtration system (without any membrane) vs. time. Crossflow data are taken from Imbrogno and Schäfer. [26] Filled

symbols indicate feed concentration values. CV: crossflow velocity. 100 ng L^{-1} E2, 1 mM $NaHCO_3$, 10 mM NaCl, pH 8.1 ± 0.2, 23 ± 1 °C. Error bars represent propagated error from operational parameter variations and analytical error.

The amount of E2 adsorbed by the filtration system in dead-end mode was low (0.15 ng cm^{-2}) and reached saturation after 50 min. The adsorbed mass by the system in different crossflow configurations was 4−8 times higher than in dead-end mode. The dampener could cause extra adsorption. Hence, the system was operated in dead-end mode and without a dampener for VaCNT membranes to minimise system adsorption.

7.3. Variation of pressure in filtration with VaCNT membranes

The pressure increased in most filtration experiments with SHs, and this increase varied between experiments. Besides, a dampener was not used, which might induce local pressure variation due to pump pulsation. Supplementary Figure 16 and Supplementary Figure 17 show the pressure data vs. time in all experiments acquired every second.

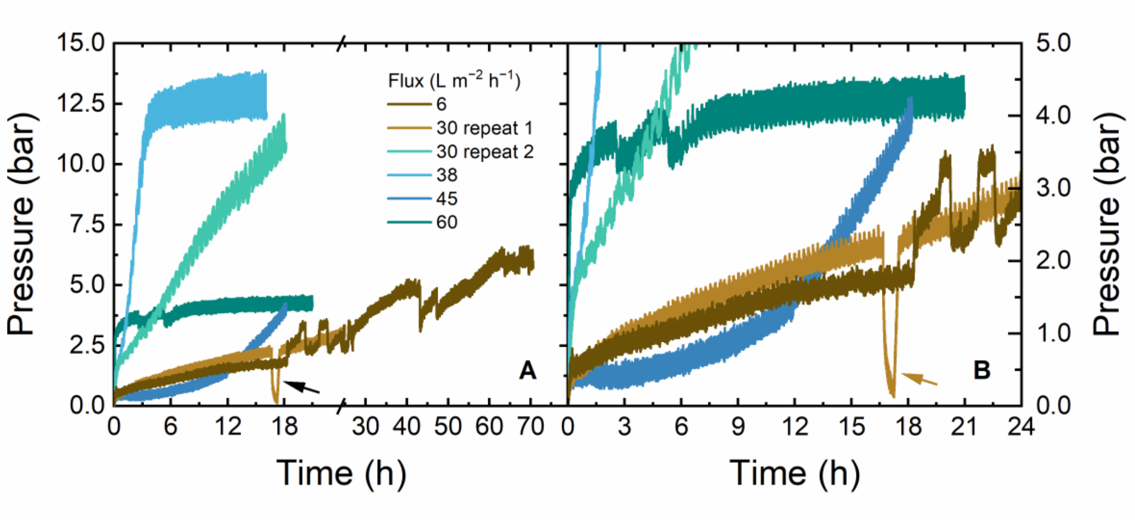

Supplementary Figure 16. A – Pressure vs. time in E2 filtration with varying flux (A). B – A zoom-in of pressure data in A in the time range 0−24 h and pressure range 0−5 bar. CNT pore diameter 1.7 nm, 100 ng L^{-1} E2, 1 mM $NaHCO_3$, 10 mM NaCl, pH 8.2 ± 0.1. Arrow indicates a temporary switch-off of the pump after the core experiment at 30 L m^{-2} h^{-1} was complete.

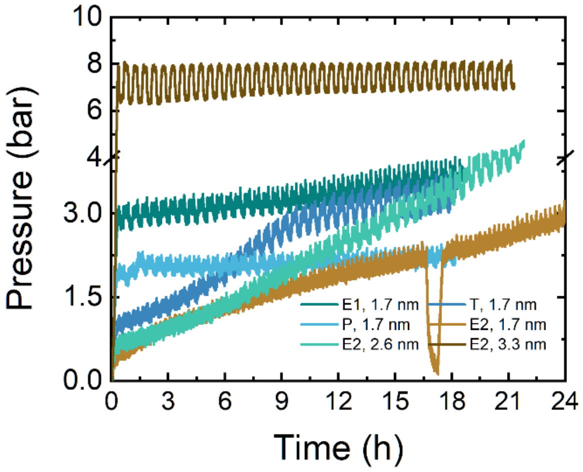

Supplementary Figure 17. A – Pressure vs. time in E2 filtration with varying SH and pore diameter. B – A zoom-in of pressure data in A in the time range 0−24 h and pressure range 0−5 bar. 100 ng L^{-1} E2, 1 mM $NaHCO_3$, 10 mM NaCl, pH 8.2 ± 0.1, 23.0 ± 0.2 °C.

The pressure fluctuation caused by pump pulsation was around 7% in most experiments, and 10% in the experiments with the 3.3 nm diameter VaCNT membrane, in which the pressure was consistently high at 7.3 ± 0.7 bar. This results in flow variation and hence variation of the drag force in the VaCNT membrane pores. In most experiments, the pressure increased, which caused a loss of membrane permeability over time.

The reasons for pressure increase in VaCNT membrane filtration experiments are illustrated schematically in Supplementary Figure 18.

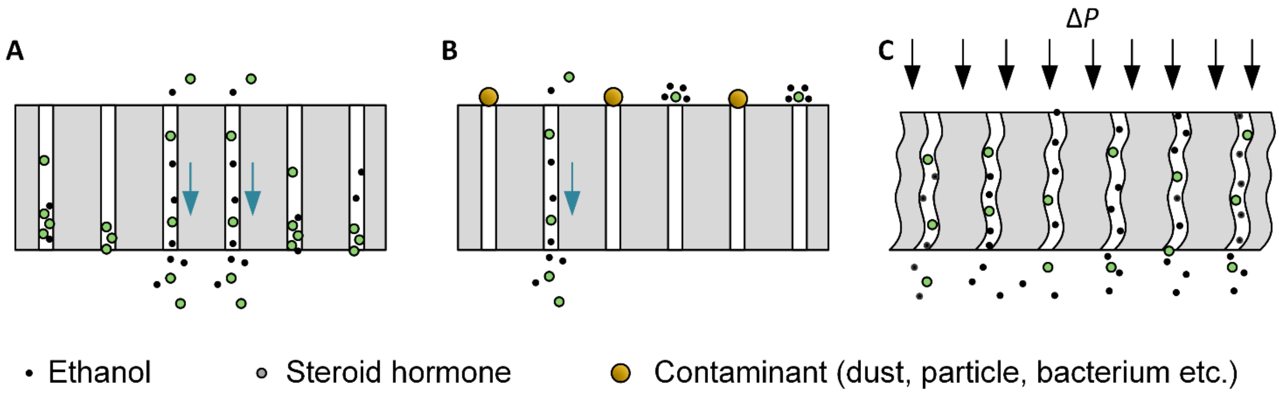

Supplementary Figure 18. Potential reasons for the pressure increase throughout the filtration experiments with VaCNT membranes. A – Internal pore blocking caused by the adsorption of SH and/or ethanol. B – External pore blocking caused by the retention of hormone−ethanol clusters and contaminants. C – VaCNT membrane deformation caused by the applied pressure. The membrane thickness, pore size, and the sizes of ethanol, SH and contaminant are not to scale.

In cases A and B, the number of conducting CNTs reduces over time, which increases the flow velocity (hence flow rate) per unblocked CNT. In case C, the number of conducting CNTs is unchanged and hence, the flow velocity is unchanged given the constant flux.

To determine which of the above cases (A, B or C) was relevant, a specific experiment was performed at around 30 L m^{-2} h^{-1} flux with a VaCNT 2.6 nm membrane coupon. Before the experiment, the system was thoroughly cleaned with bleach (calcium hypochlorite 0.5 wt.%), HCl 0.1 M, NaOH 0.1 M, warm tap water, and Milli-Q water. Filtration was firstly done with Milli-Q until 100 mL of permeate was obtained, and subsequent simulated background (1 mM $NaHCO_3$, 10 mM NaCl and 30 mg L^{-1} ethanol) until 100 mL of permeate, and then 1000 ng L^{-1} feed E2 (which includes 1 mM $NaHCO_3$, 10 mM NaCl and 300 mg L^{-1} ethanol) for 65 mL of permeate (Supplementary Figure 19).

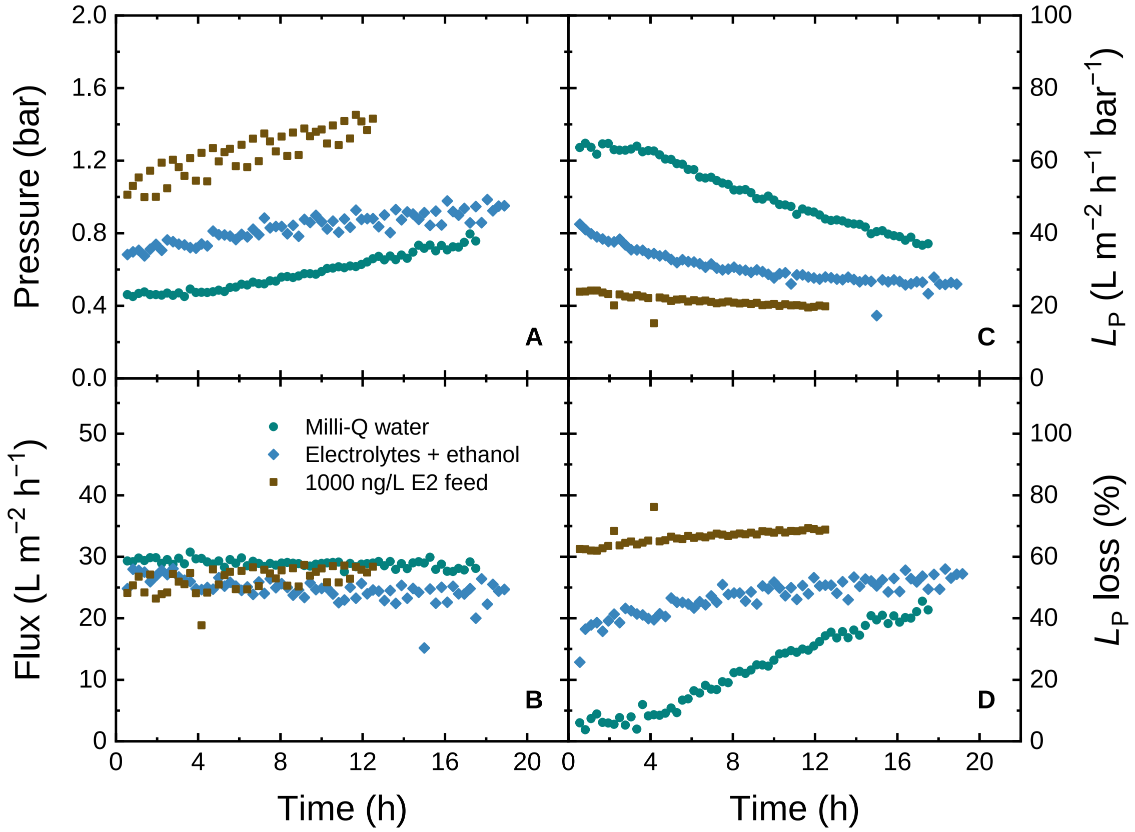

Supplementary Figure 19. Changes in pressure (A), flux (B), permeability (C), and permeability loss (D) seen with a VaCNT membrane (2.6 nm) in the filtration with Milli-Q water and subsequent simulated water matrix (1 mM $NaHCO_3$, 10 mM NaCl and 30 mg L^{-1} ethanol), and 1000 ng L^{-1} E2 solution (with 1 mM $NaHCO_3$, 10 mM NaCl and 300 mg L^{-1} ethanol). Temperature was controlled at 23.0 ± 0.2 °C.

In the first 4 h of Milli-Q water filtration, the pressure was constant at 0.45 bar. Similar stability was achieved in pure water flux tests before the SH filtration experiments. In the next 16 h of Milli-Q

water filtration, the pressure increased to 0.8 bar, resulting in a permeability loss of 42% at 18 h (so cases A and B are not likely). The membrane could be compressed because of long exposure to pressure and water (case C). In the subsequent experisments with simulated background and 1000 ng L^{-1} E2 solution, pressure increased only gradually to 1.0 and 1.5 bar, corresponding to a permeability decrease of 60% and 70% from initial pure water permeability, respectively.

A shorter filtration test (5 h) with a VaCNT 1.7 nm was performed with only Milli-Q water, and pressure and permeability data are compared with those of the above 2.6 nm membrane experiment in Supplementary Figure 20.

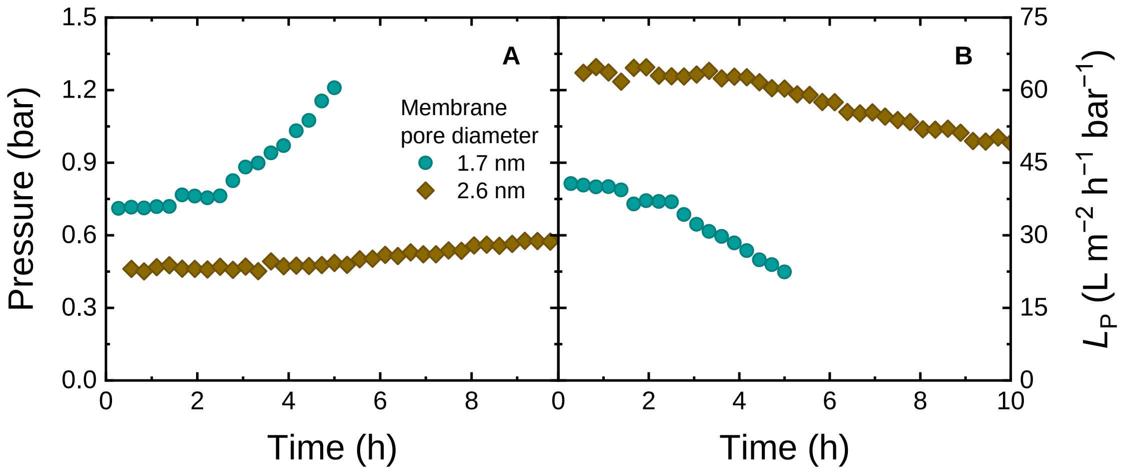

Supplementary Figure 20. Changes in pressure and permeability in Milli-Q water filtration tests with 1.7 and 2.6 nm membrane coupons. Temperature was controlled at 23.0 ± 0.2 °C.

In the first 2−4 h, the pressure was constant in both experiments, and there was no change in membrane permeability over time. After these first hours, the pressure started to increase and caused a permeability drop from 38 to 22 L m^{-2} h^{-1} bar^{-1} within 3 h and from 60 to 47 L m^{-2} h^{-1} bar^{-1} within 6 h with the tested 1.7 nm and 2.6 nm pore diameter VaCNT membranes, respectively.

The permeability loss appears to not be not directly linked to the water quality, while both membrane compression and pore blocking are possible mechanisms. The permeability loss occured for both membrane pore diameters 1.7 nm and 2.6 nm. The quantity of blocked pores could not be determined in the current experiments.

To determine any significant compression caused by the filtration, the cross-section of a VaCNT membrane piece after a filtration experiment at a pressure of up to 12 bar was visualised with helium ion microscopy, as shown in Supplementary Figure 21.

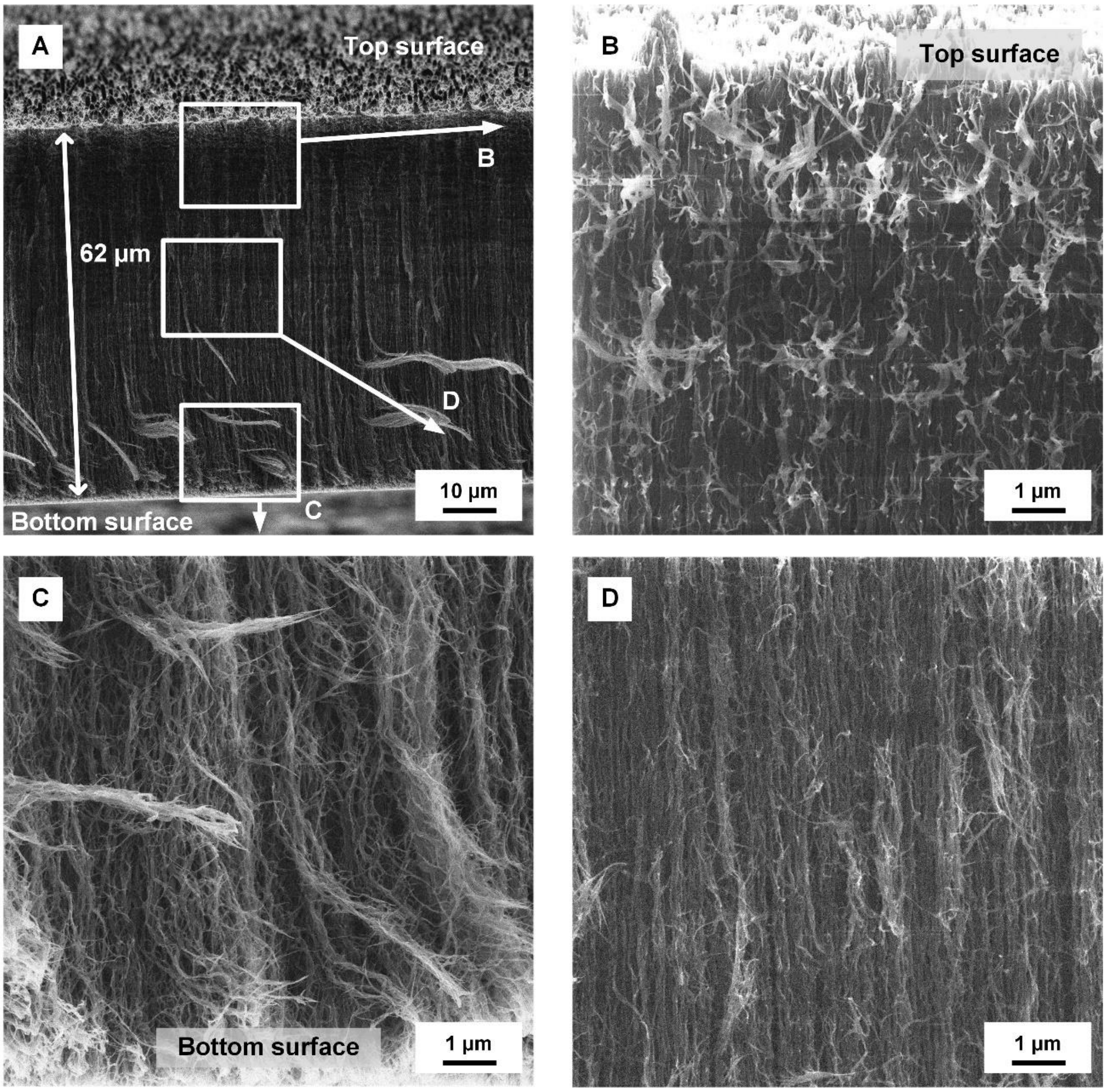

Supplementary Figure 21. A – Cross-section of a VaCNT membrane after filtration at a pressure of up to 12 bar. B, C and D – Zoom-ins of the cross-section at the top surface, at the bottom surface, and in the middle of the membrane.

From Supplementary Figure 21 A, the membrane thickness (62 µm) was similar to the value determined for the pristine membrane (57 ± 2 µm) of the same batch (Batch 3, see Table 1 in the main text). From Supplementary Figure 21 B, at the top surface, the cylindrical structures in front are zig-zag probably because of the artefacts during cross-section preparation, while the cylindrical structures in the back appeared linear. The artefacts were more prominent at the bottom surface (Supplementary Figure 21 C) preventing the observation of structures in the back. From Supplementary Figure 21 D, the cylindrical structures with very low tortuosity were clearly observed. In summary, although VaCNT membrane compression during filtration is possible, the membrane cross-section micrographs show that the compression did not significantly alter the membrane structure.

Supplementary References

1. Bocquet L., Charlaix E., Nanofluidics, from bulk to interfaces, *Chem. Soc. Rev.* **39**, 1073-1095 (2010).

2. Raviv U., Klein J., Fluidity of bound hydration layers, *Science* **297**, 1540-1543 (2002).

3. Li T.-D., Gao J., Szoszkiewicz R., Landman U., Riedo E., Structured and viscous water in subnanometer gaps, *Phys. Rev. B* **75**, 115415 (2007).

4. Rabinowitz J., Cohen C., Shepard K.L., An electrically actuated, carbon-nanotube-based Biomimetic ion pump, *Nano Lett.* **20**, 1148-1153 (2020).

5. Král P., Shapiro M., Nanotube electron drag in flowing liquids, *Phys. Rev. Lett.* **86**, 131-134 (2001).

6. Niguès A., Siria A., Vincent P., Poncharal P., Bocquet L., Ultrahigh interlayer friction in multiwalled boron nitride nanotubes, *Nat. Mater.* **13**, 688-693 (2014).

7. Semião A.J.C., Schäfer A.I., Removal of adsorbing estrogenic micropollutants by nanofiltration membranes. Part A—Experimental evidence, *J. Mem. Sci.* **431**, 244-256 (2013).

8. Cousins K., ChemOffice Plus: A package of programs for chemists, *J. Chem. Inf. Comput. Sci.* **33**, 788-789 (1993).

9. Kannam S.K., Todd B.D., Hansen J.S., Daivis P.J., How fast does water flow in carbon nanotubes?, *J. Chem. Phys.* **138**, 094701 (2013).

10. Hanasaki I., Nakatani A., Flow structure of water in carbon nanotubes: Poiseuille type or plug-like?, *J. Chem. Phys.* **124**, 144708 (2006).

11. Al Quddus N., Moussa W.A., Bhattacharjee S., Motion of a spherical particle in a cylindrical channel using arbitrary Lagrangian–Eulerian method, *J. Colloid Interface Sci.* **317**, 620-630 (2008).

12. Goldman A.J., Cox R.G., Brenner H., Slow viscous motion of a sphere parallel to a plane wall—I Motion through a quiescent fluid, *Chem. Eng. Sci.* **22**, 637-651 (1967).

13. Kendall K., Adhesion: Molecules and mechanics, *Science* **263**, 1720-1725 (1994).

14. Perez E.M., Martin N., π-π interactions in carbon nanostructures, *Chem. Soc. Rev.* **44**, 6425-6433 (2015).

15. Israelachvili J.N., Chapter 7 − Repulsive steric forces, total intermolecular pair potentials, and liquid structure, in: *Intermolecular and surface forces*, 3rd ed., Elsevier, Academic Press, 133-150 (2011).

16. Israelachvili J.N., Chapter 13 − Van der Waals forces between particles and surfaces, in: *Intermolecular and surface forces*, 3rd ed., Elsevier, Academic Press, 253-289 (2011).

17. Rajter R.F., French R.H., Ching W.Y., Podgornik R., Parsegian V.A., Chirality-dependent properties of carbon nanotubes: Electronic structure, optical dispersion properties, Hamaker coefficients and van der Waals–London dispersion interactions, *RSC Advances* **3**, 823-842 (2013).

18. Takagishi H., Masuda T., Shimoda T., Maezono R., Hongo K., Method for the calculation of the Hamaker constants of organic materials by the Lifshitz macroscopic approach with Density Functional Theory, *The Journal of Physical Chemistry A* **123**, 8726-8733 (2019).

19. Parsegian V.A., *Van der Waals forces : A handbook for biologists, chemists, engineers, and physicists*, Cambridge University Press, New York, (2006).

20. Atkins P., de Paula J., Keeler J., Molecular interactions, in: *Atkins' Physical Chemistry*, 11th ed., Oxford University Press, Oxford, England, UK, 583-637 (2018).

21. Bocquet L., Barrat J.-L., Flow boundary conditions from nano- to micro-scales, *Soft Matter* **3**, 685-693 (2007).

22. Falk K., Sedlmeier F., Joly L., Netz R.R., Bocquet L., Molecular origin of fast water transport in carbon nanotube membranes: Superlubricity versus curvature dependent friction, *Nano Lett.* **10**, 4067-4073 (2010).

23. Cieplak M., Smith E.D., Robbins M.O., Molecular origins of friction: The force on adsorbed layers, *Science* **265**, 1209 (1994).

24. Chen X., Li J., Superlubricity of carbon nanostructures, *Carbon* **158**, 1-23 (2020).

25. Nguyen M.N., Trinh P.B., Burkhardt C.J., Schäfer A.I., Incorporation of single-walled carbon nanotubes in ultrafiltration support structure for the removal of steroid hormone micropollutants, *Sep. Purif. Technol.* **264**, 118405 (2021).

26. Imbrogno A., Schäfer A.I., Micropollutants breakthrough curve phenomena in nanofiltration: Impact of operational parameters, *Sep. Purif. Technol.* **267**, 118406 (2021).

27. Jue M.L., Buchsbaum S.F., Chen C., Park S.J., Meshot E.R., Wu K.J.J., Fornasiero F., Ultra-permeable single-walled carbon nanotube membranes with exceptional performance at scale, *Adv. Sci.* **7**, 2001670 (2020).

28. Hilal N., Al-Zoubi H., Darwish N.A., Mohammad A.W., Characterisation of nanofiltration membranes using atomic force microscopy, *Desalination* **177**, 187-199 (2005).

29. Kanani D.M., Fissell W.H., Roy S., Dubnisheva A., Fleischman A., Zydney A.L., Permeability–selectivity analysis for ultrafiltration: Effect of pore geometry, *J. Mem. Sci.* **349**, 405-410 (2010).

30. Freger V., Nanoscale heterogeneity of polyamide membranes formed by interfacial polymerization, *Langmuir* **19**, 4791-4797 (2003).

31. Boussu K., Zhang Y., Cocquyt J., Van der Meeren P., Volodin A., Van Haesendonck C., Martens J.A., Van der Bruggen B., Characterization of polymeric nanofiltration membranes for systematic analysis of membrane performance, *J. Mem. Sci.* **278**, 418-427 (2006).

32. Merck Millipore, OEM membranes: Microfiltration and ultrafiltration membranes, http://www.merckmillipore.com/GB/en/products/ivd-oem-materials-reagents/oem-membranes/L.eb.qB.TUMAAAE_L_53.Lxi,nav?bd=1, accessed on 8th June 2020.

33. Bowen W.R., Welfoot J.S., Modelling of membrane nanofiltration—pore size distribution effects, *Chem. Eng. Sci.* **57**, 1393-1407 (2002).

34. Aschermann G., Jeihanipour A., Shen J., Mkongo G., Dramas L., Croue J.P., Schäfer A., Seasonal variation of organic matter concentration and characteristics in the Maji ya Chai River (Tanzania): Impact on treatability by ultrafiltration, *Water Res.* **101**, 370-381 (2016).

35. Imbrogno A., Schäfer A.I., Comparative study of nanofiltration membrane characterization devices of different dimension and configuration (cross flow and dead end), *J. Mem. Sci.* **585**, 67-80 (2019).

36. Worch E., Eine neue Gleichung zur Berechnung von Diffusionskoeffizienten gelöster Stoffe, *Vom Wasser* **81**, 289-297 (1993).

37. Deen W.M., Hindered transport of large molecules in liquid-filled pores, *AlChE J.* **33**, 1409-1425 (1987).

38. Mulder M., Characterisation of membranes, in: *Basic Principles of Membrane Technology*, Springer, Dordrecht, The Netherlands, 110-144 (1996).

39. Mattia D., Leese H., Lee K.P., Carbon nanotube membranes: From flow enhancement to permeability, *J. Mem. Sci.* **475**, 266-272 (2015).

40. Schäfer A.I., Mauch R., Waite T.D., Fane A.G., Charge effects in the fractionation of natural organics using ultrafiltration, *Environ. Sci. Technol.* **36**, 2572-2580 (2002).

41. Holt J.K., Park H.G., Wang Y., Stadermann M., Artyukhin A.B., Grigoropoulos C.P., Noy A., Bakajin O., Fast mass transport through sub-2-nanometer carbon nanotubes, *Science* **312**, 1034 (2006).

42. Joly L., Capillary filling with giant liquid/solid slip: Dynamics of water uptake by carbon nanotubes, *J. Chem. Phys.* **135**, 214705 (2011).

43. Gravelle S., Ybert C., Bocquet L., Joly L., Anomalous capillary filling and wettability reversal in nanochannels, *Phys. Rev. E* **93**, 033123 (2016).

44. Patel H., Fixed-bed column adsorption study: A comprehensive review, *Appl. Water Sci.* **9**, 45 (2019).

45. Nguyen M.N., Weidler P.G., Schwaiger R., Schäfer A.I., Interactions between carbon-based nanoparticles and steroid hormone micropollutants in water, *J. Hazard. Mater.* **402**, 122929 (2020).

46. Semião A.J.C., Foucher M., Schäfer A., Removal of adsorbing estrogenic micropollutants by nanofiltration membranes: Part B—Model development, *J. Mem. Sci.* **431**, 257-266 (2013).

47. Ugarte D., Châtelain A., de Heer W.A., Nanocapillarity and chemistry in carbon nanotubes, *Science* **274**, 1897-1899 (1996).

48. Pan X., Bao X., The effects of confinement inside carbon nanotubes on catalysis, *Acc. Chem. Res.* **44**, 553-562 (2011).

49. Johnson E.R., Keinan S., Mori-Sánchez P., Contreras-García J., Cohen A.J., Yang W., Revealing noncovalent interactions, *J. Am. Chem. Soc.* **132**, 6498-6506 (2010).

50. Liu H.-C., Fried J.R., Breakthrough of lysozyme through an affinity membrane of cellulose-cibacron blue, *AlChE J.* **40**, 40-49 (1994).

51. Merck Millipore, Durapore® membrane filter, 0.22 µm, hydrophilic PVDF, 13 mm membrane, https://www.merckmillipore.com/DE/en/product/Durapore-Membrane-Filter-0.22m,MM_NF-GVWP01300#overview, accessed on 27 April 2022.

52. Lu L., Shao Q., Huang L., Lu X., Simulation of adsorption and separation of ethanol–water mixture with zeolite and carbon nanotube, *Fluid Phase Equilib.* **261**, 191-198 (2007).

53. Falk K., Sedlmeier F., Joly L., Netz R.R., Bocquet L., Ultralow liquid/solid friction in carbon nanotubes: Comprehensive theory for alcohols, alkanes, OMCTS, and water, *Langmuir* **28**, 14261-14272 (2012).

54. Hurwitz A.R., Liu S.T., Determination of aqueous solubility and pKa values of estrogens, *J. Pharm. Sci.* **66**, 624-627 (1977).

55. Liu S.T., Carney C.F., Hurwitz A.R., Adsorption as a possible limitation in solubility determination, *J. Pharm. Pharmacol.* **29**, 319-321 (1977).

56. Shareef A., Angove M.J., Wells J.D., Johnson B.B., Aqueous solubilities of estrone, 17β-estradiol, 17α-ethynylestradiol, and bisphenol A, *J. Chem. Eng. Data* **51**, 879-881 (2006).

57. Bischoff F., Pilhorn H.R., The state and distribution of steroid hormones in biologic systems: Solubilities of testosterone, progesterone and alpha-estradiol in aqueous salt and protein solution and in serum, *J. Biol. Chem.* **174**, 663-682 (1948).

58. Eik-Nes K., Schellman J.A., Lumry R., Samuels L.T., The binding of steroids to protein. I. Solubility determinations, *J. Biol. Chem.* **206**, 411-419 (1954).

59. Kabasakalian P., Britt E., Yudis M.D., Solubility of some steroids in water, *J. Pharm. Sci.* **55**, 642 (1966).

60. Barry B.W., Eini D.I.D.E., Solubilization of hydrocortisone, dexamethasone, testosterone and progesterone by long-chain polyoxyethylene surfactants, *J. Pharm. Pharmacol.* **28**, 210-218 (1976).

61. Lange W.E., Amundson M.E., Soluble steroids I: Sugar derivatives, *J. Pharm. Sci.* **51**, 1102-1106 (1962).

62. Lata G.F., Le D.K., Steroid solubility studies with aqueous solutions of urea and ureides, *Arch. Biochem. Biophys.* **109**, 434-441 (1965).

63. Lin H.-M., Nash R.A., An experimental method for determining the Hildebrand solubility parameter of organic nonelectrolytes, *J. Pharm. Sci.* **82**, 1018-1026 (1993).

64. Fulford M.D., Slonek J.E., Groves M.J., A note on the solubility of progesterone in aqueous polyethylene glycol 400, *Drug Dev. Ind. Pharm.* **12**, 631-635 (1986).

65. Haskins A.L., Solubility of progesterone in water and in saline, *Proc. Soc. Exp. Biol. Med.* **70**, 228-229 (1949).

66. Ruchelman M.W., Solubility studies of estrone in organic solvents using gas-liquid chromatography, *Anal. Biochem.* **19**, 98-108 (1967).

67. Doisy E.A., Huffman M.N., Thayer S.A., Doisy E.A., Solubilities of some estrogens, *J. Biol. Chem.*, 283-285 (1941).

68. Ruchelman M.W., Haines P., Solubility studies of estradiol in organic solvents using gas-liquid chromatography, *J. Chromatogr. Sci.* **5**, 290-296 (1967).

69. Ruchelman M.W., Solubility studies of testosterone in organic solvents using gas chromatography, *J. Chromatogr. Sci.* **9**, 235-240 (1971).

70. Sieminska L., Ferguson M., Zerda T.W., Couch E., Diffusion of steroids in porous sol-gel glass: Application in slow drug delivery, *J. Sol-Gel Sci. Technol.* **8**, 1105-1109 (1997).

71. Harper P.M., Gani R., Kolar P., Ishikawa T., Computer-aided molecular design with combined molecular modeling and group contribution, *Fluid Phase Equilib.* **158-160**, 337-347 (1999).

72. Bui N., Meshot E.R., Kim S., Pena J., Gibson P.W., Wu K.J., Fornasiero F., Ultrabreathable and protective membranes with sub-5 nm carbon nanotube pores, *Adv. Mater.* **28**, 5871-5877 (2016).

73. Buchsbaum S.F., Jue M.L., Sawvel A.M., Chen C., Meshot E.R., Park S.J., Wood M., Wu K.J., Bilodeau C.L., Aydin F., Pham T.A., Lau E.Y., Fornasiero F., Fast permeation of small ions in carbon nanotubes, *Adv. Sci.* **8**, 2001802 (2021).

74. Alvarez S., A cartography of the van der Waals territories, *Dalton Transactions* **42**, 8617-8636 (2013).

75. Bondi A., van der Waals volumes and radii, *J. Phys. Chem.* **68**, 441-451 (1964).

76. US Environmental Protection Agency, Definition and procedure for the determination of the method detection limit, revision 2, https://www.epa.gov/sites/default/files/2016-12/documents/mdl-procedure_rev2_12-13-2016.pdf, accessed on 10 November 2021.

77. Zhang W., Furusaki S., On the evaluation of diffusivities in gels using the diffusion cell technique, *Biochem. Eng. J.* **9**, 73-82 (2001).

78. Baker R.W., Membrane transport theory, in: *Membrane technology and applications*, 3rd ed., John Wiley & Sons, Chichester, West Sussex, U.K., 15-96 (2012).

79. International Association for the Properties of Water and Steam, Release on the IAPWS formulation 2008 for the viscosity of ordinary water substance, http://www.iapws.org/relguide/visc.pdf, accessed on 31 December 2021.

80. Mills R., Self-diffusion in normal and heavy water in the range 1-45.deg, *The Journal of Physical Chemistry* **77**, 685-688 (1973).

81. Kim K.J., Fane A.G., Fell C.J.D., Suzuki T., Dickson M.R., Quantitative microscopic study of surface characteristics of ultrafiltration membranes, *J. Mem. Sci.* **54**, 89-102 (1990).

82. Nakao S.-i., Determination of pore size and pore size distribution: 3. Filtration membranes, *J. Mem. Sci.* **96**, 131-165 (1994).

83. Nguyen M.N., Hervás-Martínez R., Schäfer A.I., Organic matter interference with steroid hormone removal by single-walled carbon nanotubes - ultrafiltration composite membrane, *Water Res.* **199**, 117148 (2021).
